# Associations between sexual behaviour change in young people and decline in HIV prevalence in Zambia

**DOI:** 10.1186/1471-2458-7-60

**Published:** 2007-04-23

**Authors:** Ingvild F Sandøy, Charles Michelo, Seter Siziya, Knut Fylkesnes

**Affiliations:** 1 Centre for International Health, University of Bergen, Armauer Hansen building, N-5021 Bergen, Norway; 2 Centre for International Health, University of Bergen and Department of Community Medicine, University of Zambia, Zambia; 3 Department of Community Medicine, University of Zambia, Zambia; 4 Centre for International Health, University of Bergen, Bergen, Norway

## Abstract

**Background:**

Evidence suggests that HIV prevalence amongst young Zambians has declined recently, especially in higher-education groups. We studied trends in key sexual behaviour indicators among 15–24 year-olds from 1995 to 2003, including the associations between sexual behaviour change and education.

**Methods:**

The data stem from a series of three population-based surveys conducted in 1995 (n = 1720), 1999 (n = 1946) and 2003 (n = 2637). Logistic regression and Extended Mantel Haenszel Chi Square for linear trends were used to compare the three surveys.

**Results:**

Men and lower-education groups reported more than one sexual partner in the year immediately prior to the survey more frequently than did women and higher-education groups (p < 0.01), but these proportions declined regardless of sex and residence. Substantial delays in child-bearing were observed, particularly among higher-education and urban respondents. Condom use at least for casual sexual intercourse increased from 1995 to 2003; the level was highest among urban and higher-education groups. The number of women reporting frequent dry sex using traditional agents fell during the period. Participants from the rural area and those with less education reported more sexual experience than urban and higher-education participants in 2003. The reported number of sexual partners during the year immediately prior to the survey was a factor that reduced the association between HIV and survey times among sexually active young urban men and women.

**Conclusion:**

High risk behaviours clearly decreased, especially in higher-educated and urban groups, and there is a probable association here with the decline in HIV prevalence in the study population. Fewer sexual partners and condom use were among the core factors involved for both sexes; and for women a further factor was delayed child-bearing.

## Background

A number of studies from sub-Saharan Africa have recently reported declines in HIV prevalence among young people, which appear to be associated with changes in sexual behaviour [1-6]. Several previous studies have used mathematical models to examine the effect of behaviour changes on prevalence. The best method for obtaining indications of the effect of sexual behaviour change on the prevalence of HIV is, however, to study the inter-relation between changes in incidence and sexual behaviour within the same population; though if direct incidence measurement is not possible, prevalence among young people is commonly used as a proxy [7].

We previously reported sharp declines in HIV prevalence in selected urban and rural communities in Zambia between 1995 and 2003 on the basis of three serial cross-sectional population-based surveys [8,9]. In the 15–24 age group we found that HIV prevalence declined by 44% in the 8-year period (from 5.7% to 3.2%; p = 0.143) among rural males, by 58% (from 16.1% to 6.8%; p < 0.001) among rural females, by 54% (6.9% to 3.2%; p = 0.005) among urban males and by 44% (22.5% to 12.5%; p < 0.001) among urban females.

At the start of the HIV epidemic in sub-Saharan Africa, higher-educated groups were hit hardest [10-14], a fact attributed to more extensive travelling and greater numbers of sexual partners in this group [10,11,13]. More recent studies have, however, shown that the prevalence of HIV infection is decreasing among educated persons. In several countries the prevalence among young people with higher education is now lower than among the less educated, especially in urban areas [10,12,15,16]. We also reported particularly sharp declines in prevalence among young people with higher education (≥10 years of schooling) in the period 1995 to 2003 – 62% among urban females, 71% among urban males, 84% among rural males and 90% among rural females – whereas the prevalence was more stable among less educated respondents over the same period. Thus, from being at higher risk of HIV infection in 1995, young people with higher education were at lower risk by 2003 than those with little education [8,9].

In this study we investigated whether the observed decline in HIV prevalence among young people in selected communities in Zambia was likely to be due to behavioural changes. Furthermore, we examined how education was associated with changes in sexual behaviour.

## Methods

The first population-based survey including data on HIV prevalence in Zambia was carried out in 1995 in Chelston (urban), Lusaka, and the Kapiri Mposhi district. The methods used in these surveys are described in detail elsewhere [8,10,17-19]. The study population was selected using a stratified random cluster sampling method. Ten urban clusters were selected in Chelston and five in rural Kapiri Mposhi. The survey was repeated in 1999 and 2003 using the same procedures except that the number of rural clusters was doubled in order to detect small changes. All households in the sampled clusters were included, and all household members aged ≥15 years who were found at home were asked to participate in a structured interview and to donate saliva for an anonymous HIV test [10]. This was an open cohort. It was possible to link individuals participating in two or more of the surveys, but the long periods between surveys and the high mobility of the respondents caused problems in studying incidence (small numbers and a highly selected group); hence we used prevalence among young people as a proxy of incidence. The data were double-entered in EpiInfo and validated.

### Laboratory analysis

In the first survey all the saliva samples were tested using Gacelisa HIV 1&2 (Welcome Diagnostics). The Gacelisa saliva test was validated against serum tests on paired samples from 494 samples from antenatal clinic attendees, and the specificity and sensitivity of the saliva test was 100%. Four hundred and fifty randomly selected saliva samples from the survey were also tested with Bionor HIV-1&2 (BIONOR AS), and the two saliva tests showed 99.8% agreement [11,17]. In the two follow-up surveys only Bionor HIV-1&2 (BIONOR AS) for saliva was used [10]. Those respondents who wanted to know their results also had a serum test, and this provided an extra opportunity for validation. In cases where saliva and serum results were discordant, the serum result was considered final. In all the survey rounds, 10% of negative and 10% of positive samples were re-tested by a different person.

### Data analysis

The analyses were performed using STATA version 9 and were restricted to adults aged 15–24 years. Data from the three cross-sectional surveys were compared using logistic regression, adjusting for the cluster effect and age, and Extended Mantel Haenszel Chi Square for linear trends (the latter analysis using ). Adjustment was made for age, which was considered as a continuous factor. The median age at sexual debut was calculated using survival analysis, and a log rank test for equality of survivor curves was used to compare the median ages in 1999 and 2003. (Questions about age at sexual debut, abstinence and current contraceptive use were not included in 1995, so we were only able to examine changes related to these variables between 1999 and 2003).

The total number of respondents was used as denominator when calculating the proportions for the following questions: 'ever had sex', 'sex by age 15', 'median age at debut', and 'ever given birth'. For questions about condom use, 'ever' and 'at last sexual intercourse prior to the survey', and about 'current contraceptive use', '≥2 partners during year prior to the survey', 'any casual partners during year prior to the survey', 'frequent dry sex with traditional agents', and 'self-reported experience of STI during year prior to the survey', the denominator was the number of sexually active respondents during the year prior to the survey. 'Condom use at last casual sexual intercourse' was calculated using the number of respondents who had had at least one casual partner in the course of the year prior to the survey as the denominator.

To allow comparisons to be made with our previously published analysis of prevalence changes by educational attainment in young people, the analyses were repeated with the same educational groups (0–7, 8–9 and ≥10 years of schooling) [8,9]. Analyses of the categories 'ever given birth' and 'any casual partner last year' were stratified by marital status. Logistic regression was performed with both survey time (stratified by educational level) and education (stratified by survey time) as exposure variables.

HIV prevalence declined significantly among sexually active young urban men and women and among rural women from 1995 to 2003 but not among rural men (AOR 0.51 [0.18–1.47]). To determine which behaviours contributed to the decline in HIV for urban men and women and rural women, we constructed a model based on the forward logistic regression of HIV risk by survey time, adjusted for age. We checked whether the following behaviour variables were confounders for the association between survey times and HIV among men and women: 'number of sexual partners last year' (continuous variable), 'any casual partners last year', and condom use 'ever' and 'at last sexual intercourse'. In addition we tested whether 'condom use at last casual sexual intercourse' and 'STI last year' were confounders among males; and for females we also tested the variables 'ever given birth' and 'frequent dry sex using traditional agents' (though not, in the case of women, 'condom use at last casual sexual intercourse' since <10% reported a casual partner). Variables that resulted in both a change in likelihood ratio chi square of > |3.84| (p < 0.05), and AOR for HIV comparing 2003 and 1995 closer to 1, were considered to be confounders. The confounder that gave the largest change in AOR was added to the model (by adjusting for this variable) before the process was repeated to look for additional confounders. Step by step, the model was thus expanded until no additional confounding could be detected.

Similarly, we tested how the association between survey time (2003 compared to 1995) and the odds of previous child bearing was affected when adjusting for 'condom use at last sexual intercourse', 'condom use ever', 'sexual activity last year' and 'current use of modern contraceptives' among young urban women. For rural women the proportion that had 'ever given birth' did not change significantly from 1995 to 2003 so these analyses were not performed for this group.

### Ethical aspects

The protocol was approved by the National AIDS Research Committee for the two first surveys and by the University of Zambia Research Ethics Committee for the survey in 2003. All respondents were informed that the interview was anonymous and that the saliva-based HIV test would only be used for research purposes. Informed consent was required of all participants. Everyone was offered voluntary counselling and testing free of charge using blood specimens, as required by the national guidelines for HIV testing [19].

## Results

Interview information (including sex, age and residence) was available from 1720 adults aged 15–24 years in 1995, 1946 in 1999 and 2637 in 2003. Saliva-based HIV test results were obtained from 1547 respondents in 1995, 1722 in 1999 and 2228 in 2003. In the whole sample the most common reason for not being interviewed was absence because of school, admission to hospital or temporary travel. Altogether, 16% were absent in 1995, 28% in 1999 and 20% in 2003. In all the three surveys, eligible men were approximately twice as likely to be absent as women; only 1–2% refused to be interviewed, and fewer than 10% refused to give saliva for testing [8,10,18]. The refusal rates were similar for men and women. There were no marked differences in non-response by age throughout the period, but an increase was observed in the median age of rural men successfully interviewed, from 26 in 1995 to 28 in 2003. The mean number of school years also increased somewhat for young urban male and female respondents (see Additional file 1).

**Figure 1:**
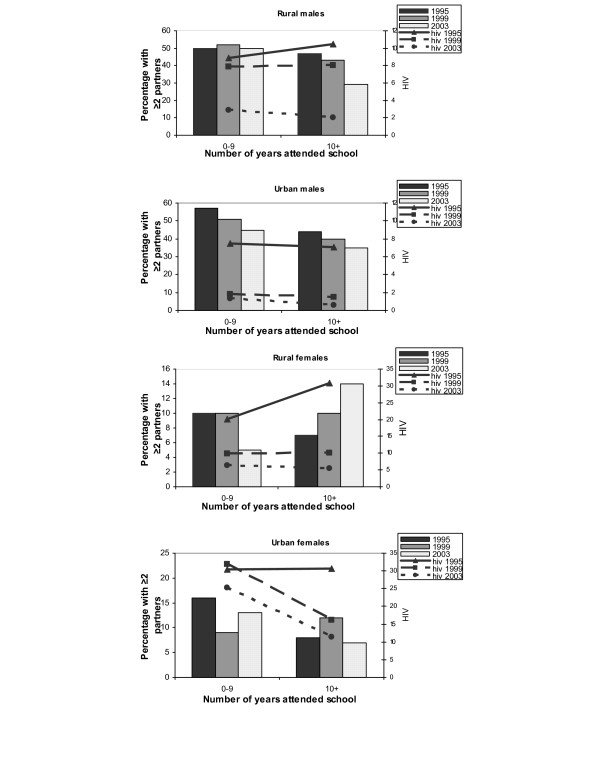
Proportion of sexually active men and women aged 15–24 with ≥2 sexual partners last 12 months and HIV prevalence among sexually active young people by educational attainment, 1995–2003.

The proportion of young respondents admitting to having had a casual partner or having had more than one partner during the year prior to the survey ranged between 39 and 52% among men and between 6 and 17% among women in both the rural and urban areas over the study period (Table 1). Fewer married than single young respondents had had casual partners, except among urban men (see Additional file 2). In addition, young people with little education were more likely to have had more than one partner during the year prior to the survey than those with more than 9 years of schooling, except among rural women (Figure 1). The proportion of respondents with more than one partner declined significantly for urban males and rural females (Table 1).

**Table 1:** Trends in HIV and key sexual behaviours among young people aged 15–24, 1995–2003.

**Indicator**	**Year**	**Rural**													
		**M**							**F**						

		**%**	**N**	**Crude OR**	**95% CI**	**AOR**	**95% CI**	**P**	**%**	**N**	**Crude OR**	**95% CI**	**AOR**	**95% CI**	**P**

**HIV infection**	1995	5.7	176	Ref.		Ref.		0.143	16.1	236	Ref.		Ref.		**<0.001**
	1999	7.5	268	1.42	0.66–3.05	1.45	0.66–3.17		10.3	380	**0.59**	**0.38–0.92**	**0.60**	**0.37–0.96**	
	2003	3.2	309	0.56	0.26–1.20	0.56	0.25–1.26		6.8	456	**0.38**	**0.19–0.78**	**0.36**	**0.19–0.67**	
															
**Ever sex**	1999	90	290	Ref.		Ref.			86	424	Ref.		Ref.		**-**
	2003	77	328	**0.37**	**0.24–0.58**	**0.32**	**0.18–0.56**		80	486	0.65	0.41–1.03	**0.53**	**0.32–0.89**	
															
**Casual partner past year**	1995	46	142	Ref.		Ref.		0.852	12	184	Ref.		Ref.		**0.023**
	1999	52	206	1.27	0.68–2.37	1.25	0.65–2.40		12	340	0.99	0.55–1.77	0.90	0.48–1.69	
	2003	48	191	1.07	0.70–1.64	1.08	0.69–1.69		7	343	**0.53**	**0.31–0.90**	**0.52**	**0.29–0.93**	
															
**>2 sexual partners past 12 months**	1995	50	147	Ref.		Ref.		0.491	10	189	Ref.		Ref.		0.056
	1999	51	206	1.03	0.74–1.43	1.02	0.72–1.42		10	340	1.03	0.54–1.95	0.95	0.49–1.85	
	2003	47	194	0.87	0.55–1.37	0.87	0.55–1.38		6	340	**0.56**	**0.34–0.92**	**0.56**	**0.33–0.96**	
															
**Ever used condom**	1995	54	147	Ref.		Ref.		0.891	26	189	Ref.		Ref.		0.098
	1999	60	207	1.25	0.73–2.14	1.32	0.74–2.36		36	338	1.57	0.82–3.02	1.59	0.85–3.00	
	2003	56	199	1.06	0.66–1.69	1.05	0.66–1.65		34	348	1.48	0.97–2.27	1.48	0.97–2.26	
															
**Used condom at last sexual intercourse**	1995	38	136	Ref.		Ref.		0.681	13	159	Ref.		Ref.		0.108
	1999	26	181	0.58	0.23–1.46	0.56	0.22–1.44		19	268	1.63	0.45–5.99	1.58	0.43–5.84	
	2003	35	133	0.91	0.48–1.73	0.93	0.49–1.77		19	248	1.67	0.86–3.24	1.69	0.87–3.29	
															
**Used condom at last casual sexual intercourse**	1995	27	62	Ref.		Ref.		0.571	9	23	Ref.		Ref.		**0.004**
	1999	14	110	**0.42**	**0.18–0.998**	0.43	0.18–1.05		2	40	0.27	0.04–1.91	0.26	0.04–1.83	
	2003	29	89	1.09	0.35–3.36	1.09	0.35–3.42		39	23	**6.75**	**2.15–21.2**	**6.31**	**1.50–26.5**	
															
**Modern contraceptive use**	1999	-	-	-	-	-	-		16	395	Ref.		Ref.		-
	2003	-	-	-	-	-	-		17	400	1.04	0.65–1.66	0.97	0.61–1.54	
															
**Ever given birth**	1995	-	-	-	-	-	-		54	257	Ref.		Ref.		0.114
	1999	-	-	-	-	-	-		61	411	1.32	0.69–2.53	1.61	0.74–3.51	
	2003	-	-	-	-	-	-		61	467	1.32	0.87–1.99	1.35	0.78–2.33	
															
**Most often traditional agents before sex**	1995	-	-			-		-	36	188	Ref.		Ref.		**<0.001**
	1999	-	-			-	-		20	381	**0.44**	**0.24–0.81**	**0.48**	**0.25–0.90**	
	2003	-	-			-	-		16	364	**0.34**	**0.16–0.71**	**0.33**	**0.15–0.72**	
															
**Indicator**	**Year**	**Urban**													

		**M**							**F**						

		**%**	**N**	**Crude OR**	**95% CI**	**AOR**	**95% CI**	**P**	**%**	**N**	**Crude OR**	**95% CI**	**AOR**	**95% CI**	**P**

**HIV infection**	1995	6.9	434	Ref.		Ref.		**0.005**	22.5	702	Ref.		Ref.		**<0.001**
	1999	7.4	432	1.07	0.67–1.72	1.01	0.61–1.66		18.3	641	**0.77**	**0.59–0.99**	**0.72**	**0.55–0.93**	
	2003	3.2	623	**0.45**	**0.28–0.70**	**0.40**	**0.25–0.66**		12.5	840	**0.49**	**0.36–0.66**	**0.43**	**0.33–0.57**	
															
**Ever sex**	1999	65	496	Ref.		Ref.		-	65	723	Ref.		Ref.		**-**
	2003	60	695	0.81	0.61–1.07	**0.70**	**0.52–0.96**		55	958	**0.65**	**0.50–0.83**	**0.54**	**0.42–0.68**	
															
**Casual partner past year**	1995	46	254	Ref.		Ref.		0.436	12	433	Ref.		Ref.		0.841
	1999	46	207	0.99	0.70–1.42	0.98	0.69–1.40		17	336	1.56	0.94–2.59	1.59	0.96–2.62	
	2003	43	244	0.87	0.63–1.20	0.86	0.63–1.17		11	363	0.93	0.66–1.30	1.00	0.73–1.37	
															
**>=2 sexual partners past 12 months**	1995	52	269	Ref.		Ref.		**0.003**	13	432	Ref.		Ref.		0.117
	1999	44	207	0.73	0.54–1.00	**0.68**	**0.48–0.99**		10	338	0.76	0.47–1.23	0.77	0.48–1.22	
	2003	39	243	0.59	0.48–0.72	**0.54**	**0.42–0.70**		10	359	0.71	0.42–1.19	0.80	0.51–1.26	
															
**Ever used condom**	1995	70	273	Ref.		Ref.		**<0.001**	59	445	Ref.		Ref.		**<0.001**
	1999	84	207	**2.22**	**1.53–3.24**	**1.82**	**1.23–2.68**		75	338	**2.09**	**1.27–3.43**	**2.08**	**1.27–3.40**	
	2003	84	263	**2.24**	**1.63–3.06**	**1.68**	**1.13–2.49**		81	387	**2.93**	**2.00–4.28**	**2.76**	**1.89–4.04**	
															
**Used condom at last sexual intercourse**	1995	53	247	Ref.		Ref.		**<0.002**	36	412	Ref.		Ref.		**<0.001**
	1999	26	255	**0.31**	**0.21–0.48**	**0.31**	**0.21–0.48**		25	425	**0.60**	**0.44–0.81**	**0.58**	**0.42–0.79**	
	2003	67	249	**1.80**	**1.04–3.11**	1.59	0.97–2.60		57	347	**2.34**	**1.45–3.79**	**2.43**	**1.51–3.93**	
															
**Used condom at last casual sexual intercourse**	1995	50	108	Ref.		Ref.		**0.023**	46	50	Ref.		Ref.		**<0.001**
	1999	65	94	**1.85**	**1.07–3.19**	**1.69**	**1.05–2.74**		53	57	1.30	0.62–2.76	1.29	0.60–2.78	
	2003	65	104	**1.89**	**1.10–3.23**	**1.68**	**1.00–2.82**		82	40	**5.53**	**2.89–10.6**	**5.80**	**2.88–11.7**	
															
**Modern contraceptive use**	1999	**-**	**-**	Ref.		Ref.			27	616	Ref.		Ref.		**-**
	2003	-	-	-	-	-	-		37	494	**1.64**	**1.13–2.37**	1.30	0.94–1.81	
															
**Ever given birth**	1995	**-**	**-**	Ref.		Ref.		**-**	39	772	Ref.		Ref.		**<0.001**
	1999	-	-	-	-	-	-		27	713	**0.57**	**0.44–0.75**	**0.43**	**0.31–0.61**	
	2003	-	-	-	-	-	-		26	834	**0.54**	**0.35–0.83**	**0.33**	**0.20–0.55**	
															
**Most often traditional agents before sex**	1995	**-**	**-**	Ref.		Ref.		**-**	20	429	Ref.		Ref.		**<0.001**
	1999	-	-	-	-	-	-		2	590	**0.09**	**0.03–0.24**	**0.10**	**0.04–0.29**	
	2003	-		-	-	-	-		5	427	**0.23**	**0.09–0.55**	**0.21**	**0.09–0.49**	

The proportions of 'condom use ever', 'condom use at last sexual intercourse' and 'condom use at last casual sexual intercourse', were highest among urban men, followed by urban women, and increased for all young adults. The most significant changes were found in the urban area, where 57% of young women reported using a condom at their last sexual intercourse in 2003 compared to 36% in 1995 (Table 1). For young rural men there was no increase. Young urban women used condoms more consistently with casual than with other partners, but for young urban men there was no difference. Individuals with higher education were more likely than the least educated to report condom use at both their last sexual intercourse prior to the survey (Table 2) and their last casual sexual encounter. Condom use also increased more significantly among high-education groups, especially educated rural women, who had lagged behind in 1995 (22% condom use at last intercourse in 1995 compared to 70% in 2003; p = 0.009). Among people with little education, condom use at last sexual intercourse was more common in the urban than the rural areas throughout the period surveyed (see Additional file 3).

**Table 2:** Proportions reporting condom use at last sexual intercourse by educational attainment among adults aged 15–24, 1995–2003. Urban and rural respondents have been pooled.

		**Males**						**Females**					
**Year**	**School years**	**%**	**N**	**Crude OR**	**95% CI**	**AOR**	**95% CI**	**%**	**N**	**Crude OR**	**95% CI**	**AOR**	**95% CI**

**1995**	***0–7***	31	142	Ref.		Ref		21	261	Ref.		Ref	
	***8–9***	50	118	**2.23**	**1.22–4.08**	**2.38**	**1.20–4.70**	32	173	**1.79**	**1.09–2.96**	**1.79**	**1.08–2.97**
	***10+***	63	122	**3.81**	**2.25–6.46**	**3.65**	**2.06–6.47**	41	133	**2.56**	**1.13–5.79**	**2.54**	**1.13–5.70**
													
**1999**	***0–7***	25	164	Ref.		Ref		21	349	Ref.		Ref	
	***8–9***	27	101	1.09	0.66–1.82	1.13	0.66–1.94	18	146	0.86	0.52–1.42	0.87	0.52–1.44
	***10+***	26	166	1.05	0.63–1.76	1.02	0.59–1.75	29	197	**1.54**	**1.08–2.19**	**1.57**	**1.09–2.25**
													
**2003**	***0–7***	28	109	Ref.		Ref		20	262	Ref.		Ref	
	***8–9***	50	72	**2.52**	**1.46–4.34**	**2.60**	**1.45–4.67**	45	112	**3.26**	**2.15–4.93**	**3.84**	**2.46–5.99**
	***10+***	73	201	**6.68**	**3.41–13.1**	**7.20**	**3.56–14.5**	64	219	**7.30**	**4.26–12.5**	**9.19**	**5.52–15.3**

**Figure 2:**
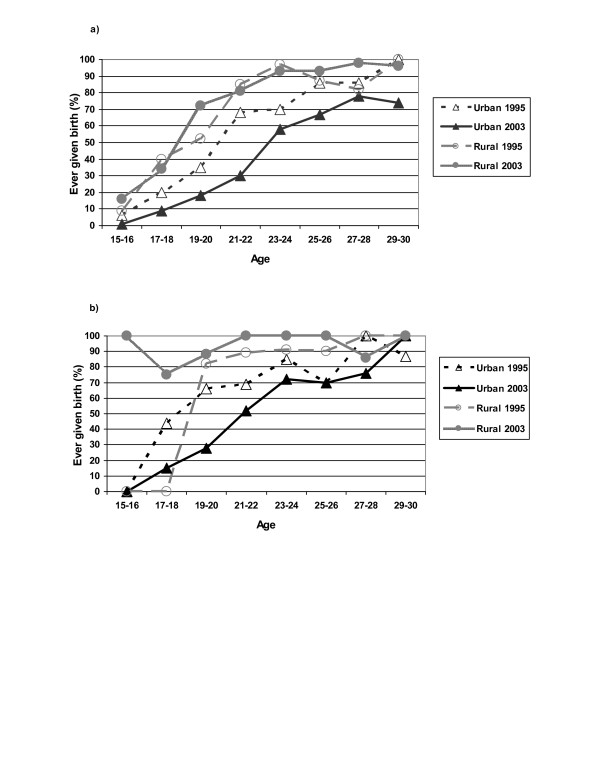
a: Proportion ever given birth among HIV-negative women. b: Proportion ever given birth among HIV-positive women.

In 1995, the proportion that had 'ever given birth' was higher among single women with higher education than among the less educated single women; however, the figure declined in the former group and remained stable in the latter up to 2003, resulting in a reversed relationship. Among married urban women with higher education the proportion that had given birth also declined, but among less educated married women and married rural women with higher education there were no significant changes (Table 3). Throughout the period, previous child-bearing was less common for young women in the urban than in the rural area. The declining proportion of women who had given birth among urban and higher-education groups was also observed among both HIV positive and HIV-negative women (Figure 2 and Additional file 4). (Note that for HIV positive rural women the sample was very small so the apparent increase in the proportion who had ever given birth was not significant.) Previous child-bearing was associated with an approximately double risk of acquiring HIV infection for single women. For married women, the risk of HIV was non-significantly higher or equal for those who had ever given birth compared to those who had not (see Additional file 5). 'Condom use at last sexual intercourse', 'condom use ever', 'no sexual activity during the last year', and 'current use of modern contraceptives' were all identified as confounding factors for the changes in the proportion of respondents who had 'ever given birth' among young urban women (see Additional file 6).

**Table 3:** Changes in the proportion of women aged 15–24 reporting who had ever given birth by educational attainment, 1995–2003

		**Rural**											
	**Marital status**	**Single**						**Married**					

**School years**	**Year**	**%**	**N**	**Crude OR**	**95% CI**	**AOR**	**95% CI**	**%**	**N**	**Crude OR**	**95% CI**	**AOR**	**95% CI**

**0–7**	*1995*	17	72	Ref.		Ref		82	99	Ref.		Ref	
	*1999*	17	94	1.03	0.43–2.44	1.38	0.72–2.66	79	214	0.83	0.26–2.66	1.02	0.26–3.94
	*2003*	18	94	1.10	0.44–2.77	1.47	0.66–3.29	86	229	1.42	0.68–2.94	1.40	0.71–2.77
													
**8–9**	*1995*	20	35	Ref.		Ref		95	19	Ref.		Ref	
	*1999*	26	31	1.39	0.88–2.19	**1.87**	**1.36–2.58**	86	29	0.35	0.04–2.78	0.19	0.01–2.72
	*2003*	24	49	1.30	0.57–2.95	2.53	0.99–6.44	89	28	0.46	0.08–2.72	0.17	0.02–1.25
													
**10+**	*1995*	29	7	Ref.		Ref		80	10	Ref.		Ref	
	*1999*	29	14	1	0.48–2.07	1.11	0.59–2.09	75	4	0.75	0.03–20.3	16.3	0.25–1056
	*2003*	14	36	**0.40**	**0.17–0.93**	**0.33**	**0.19–0.58**	89	9	2	0.12–32.5	3.97	0.29–54.9
													

		**Urban**											

		**Single**						**Married**					

**School years**	**Year**	**%**	**N**	**Crude OR**	**95% CI**	**AOR**	**95% CI**	**%**	**N**	**Crude OR**	**95% CI**	**AOR**	**95% CI**

**0–7**	*1995*	15	197	Ref.		Ref		87	71	Ref.		Ref	
	*1999*	11	141	0.74	0.36–1.55	0.52	0.23–1.17	89	37	1.20	0.41–3.53	1.12	0.42–3.00
	*2003*	14	117	0.92	0.38–2.22	0.55	0.21–1.49	79	39	0.56	0.07–4.33	0.52	0.07–3.96
													
**8–9**	*1995*	27	187	Ref.		Ref		86	56	Ref.		Ref	
	*1999*	20	161	0.68	0.39–1.19	**0.40**	**0.19–0.86**	80	35	0.67	0.24–1.88	0.43	0.11–1.68
	*2003*	18	125	0.59	0.32–1.08	**0.33**	**0.13–0.85**	83	36	0.83	0.28–2.47	0.55	0.11–2.63
													
**10+**	*1995*	22	176	Ref.		Ref		86	44	Ref.		Ref	
	*1999*	12	284	**0.51**	**0.32–0.82**	0.57	0.31–1.03	74	31	0.45	0.17–1.20	0.41	0.15–1.10
	*2003*	11	432	**0.44**	**0.28–0.69**	**0.47**	**0.29–0.78**	75	61	**0.48**	**0.24–0.98**	**0.35**	**0.17–0.69**

There was a marked reduction from 1995 to 2003 in the proportion of women who reported 'frequent dry sex using traditional agents' in both the urban and rural areas (Table 1). The practice was more common in the least educated groups throughout the period, but decreased for all educational levels (see Additional file 7 and 8). Use of modern contraceptives (condom, pill, injections, IUD) was more common among urban and among more highly educated females. It increased between 1999 and 2003 for rural and urban females with higher education and urban females with the lowest educational attainment (from 19% to 35% among urban women with 0–7 years of schooling) (see Additional file 9 and 10). Interestingly, in 1999, HIV prevalence among women currently using modern contraceptives was more than twice as high as among those who did not use modern contraceptives (AOR 2.49 [95% CI 1.47–4.21] for young urban females and AOR 2.57 [1.62–4.09] for young rural females), but in 2003 this association was not significant (AOR 0.92 [0.61–1.38] and AOR 0.77 [0.27–2.22], respectively). When we distinguished between condoms and the three other modern contraceptive methods, we found that condom use was a risk factor for HIV infection among urban women in 1999, and use of IUDs, contraceptive pills or injections was a risk factor among rural women in 1999, but neither of these associations was significant in 2003.

There was a significant drop between 1999 and 2003 in the proportion of rural respondents and high-education urban respondents who reported ever having had sex (Table 1 and Additional file 11). The median age at sexual debut increased among higher-education groups of rural men and urban women (see Additional file 12). The proportion with sexual experience was lower among urban than among rural men and women, and lower for those with more than 9 years of schooling compared to the least educated (67% vs. 84% of women in the rural area in 2003; AOR 0.13: 0.07–0.26; other results not shown). Interestingly, comparisons of the proportions of reported early sexual debut between 1999 and 2003 showed significant differences among young rural men and urban women. In 1999, 15% of urban female and 42% of rural male 15–19 year-olds reported sex before age 15, but 4 years later, in 2003, 5% of urban female and 24% of rural male 19–23 year-olds reported the same (see Additional file 13). For older men and women the differences in reporting were not significant (see Additional file 14). We also found that up to 8% of young people who claimed to be virgins were in fact HIV positive in 1999, and none of these reported having received a blood transfusion (see Additional file 15).

The risk of acquiring HIV infection in 2003 compared to 1995 (AOR) among sexually active young people was 0.55 [95%CI 0.31–0.96] for urban males, 0.35 [0.28–0.45] for urban females, and 0.27 [95%CI 0.11–0.68] for rural females. Using the survey time as exposure in modelling, we found that 'number of sexual partners during the past year', 'any casual partners during the past year', 'condom use at last sexual intercourse',' condom use at last casual sexual intercourse' and 'condom use ever' were all confounders for the association between HIV and survey time among sexually active young urban men. After adjustment for 'condom use at last casual sexual intercourse', there was no significant difference in the risk of HIV between 2003 and 1995 (Table 4). 'Ever given birth', 'number of sexual partners past year', 'any casual partners past year', 'condom use at last sexual intercourse', 'condom use last casual sexual intercourse', 'condom use ever' and 'frequent dry sex using traditional agents' were confounders for the association between HIV and survey time among sexually active young urban women. After adjusting the association between HIV and survey time for 'any casual partners past year' and 'ever given birth', the association between HIV and survey time was still significant, and no new confounders were found (Table 5). Among rural women, 'ever condom use' was the only confounder, and after adjusting for this variable the association between HIV and survey time was still significant (Table 6).

**Table 4:** Age-adjusted odds ratio (AOR) of risk of HIV by survey time comparing 2003 and 1995, adjusting for sexual behaviour variables, for urban men aged 15–24

One by one behaviour indicator included			
**Behaviour indicator**	**Chi-square change**	**AOR**	**95% CI**

Number of sex partners past 12 months**	**-15.0**	**0.72**	**0.36–1.42**
Casual partner past year	**-20.59**	**0.66**	**0.27–1.64**
Condom use at last sexual intercourse	**-18.77**	**0.70**	**0.38–1.27**
Ever used condom	**-19.06**	**0.68**	**0.40–1.16**
Used condom at last casual sexual intercourse	**-21.07**	**1.12**	**0.71–1.77**
STI past year*	21.98	0.55	0.32–0.94

Notes: **Before adding the behaviour variables the Chi square was 22.19 and AOR 0.55 (0.31–0.96) in 2003 compared to 1995**. If the chi square changed > |3.84|, the added variable was a confounding variable. The denominator was the number of sexually active respondents during the year prior to the survey. *The Chi square change was significant, but the AOR was unchanged. **The chief confounder in this step.

**Table 5:** Age-adjusted odds ratio (AOR) of risk of HIV by survey time comparing 2003 and 1995, adjusting for sexual behaviour variables, for urban women aged 15–24

One by one behaviour indicator included				Two behaviour variables included				Three behaviour variables included			
**Beh. indicator**	**Chi-square change**	**AOR**	**95% CI**	**Beh. indicator**	**Chi-square change**	**AOR**	**95% CI**	**Beh. indicator**	**Chi-square change**	**AOR**	**95% CI**

Ever given birth	**-4.51**	**0.49**	**0.37–0.63**	Casual partner and ever given birth**	**-8.34**	**0.59**	**0.42–0.83**	-			
Number of sex partners past 12 months	**-26.97**	**0.52**	**0.38–0.70**	Casual partner and number of sex partners	-1.38	0.53	0.38–0.73	Casual partner, ever given birth and number of sexual partners	1.62	0.58	0.41–0.82
Casual partner past year**	**-27.25**	**0.54**	**0.39–0.75**	-				-			
Condom use at last sexual intercourse	**6.57**	**0.45**	**0.32–0.63**	Casual partner and condom use at last sexual intercourse	13.53	0.46	0.34–0.63	Casual partner, ever given birth and condom use at last sexual intercourse*	16.44	0.52	0.37–0.72
Ever used condom	**-18.06**	**0.50**	**0.37–0.68**	Casual partner and ever used condom	3.37	0.51	0.37–0.68	Casual partner, ever given birth and ever used condom*	4.32	0.56	0.41–0.76
Frequent dry sex	**-30.29**	**0.49**	**0.37–0.64**	Casual partner and frequent dry sex	**-8.85**	**0.54**	**0.38–0.77**	Casual partner, ever given birth and frequent dry sex#	-5.83	0.59	0.42–0.83

Notes: **Before adding the behaviour variables the Chi square was 53.88 and AOR 0.35 (0.28–0.45) in 2003 compared to 1995**. If the chi square changed > |3.84|, the added variable was a confounding variable. The denominator was the number of sexually active respondents during the year prior to the survey.

*The Chi square change was significant, but the AOR was further from 1, which meant that adjusting for 'condom use at last sexual intercourse' or 'ever condom use' in addition to 'any casual partners past year' increased the strength of the association between HIV and survey time, rather than reducing it. # The Chi square change was significant, but the AOR was unchanged. **The chief confounder in this step.

**Table 6:** Age-adjusted odds ratio (AOR) of risk of HIV by survey time comparing 2003 and 1995, adjusting for sexual behaviour variables, for rural women aged 15–24

One by one behaviour indicator included			
**Beh. Indicator**	**Chi-square change**	**AOR**	**95% CI**

Ever given birth	0.15	0.28	0.11–0.72
Number of sex partners past 12 months	-1.7	0.31	0.13–0.75
Casual partner past year	-0.62	0.30	0.12–0.72
Condom use at last sexual intercourse	-3.45	0.28	0.09–0.81
Ever used condom**	**-4.81**	**0.32**	**0.13–0.75**
Frequent dry sex	-1.9	0.26	0.11–0.63

Notes: **Before adding the behaviour variables the Chi square was 9.53 and AOR 0.27 (0.11–0.68) in 2003 compared to 1995**. If the chi square changed > |3.84|, the added variable was a confounding variable. The denominator was the number of sexually active respondents during the year prior to the survey. **The chief confounder in this step.

We also carried out similar modelling for higher-education groups only. We still found that the strongest confounder was 'condom use at last casual sexual intercourse' for urban men and 'condom use ever' for rural women. However, for urban women with higher education, all the same indicators were confounders as for all urban women, but 'frequent dry sex' was the strongest. For rural men, adjusting for 'any casual partners past year' and 'condom use at last sexual intercourse' changed the AOR from 0.24 [0.13–0.42] to 0.70 [0.49–0.99] (see Additional file 16).

## Discussion

The findings showed a decline in high risk sexual behaviour and an increase in reported condom use among young adults aged 15–24 years. These changes were most prominent in the higher-education groups, and included fewer sexual partners and increased use of condoms at last sexual intercourse; and among women, delayed child-bearing and less use of traditional agents before sex. We have already reported a marked decline in HIV prevalence, especially among young people with higher education, in the same surveys [8,9]. Our modelling of possible confounders of the association between survey time and HIV prevalence showed that differences in the above-mentioned indicators contributed to the decline in HIV prevalence among sexually active young people between 1995 and 2003. Mortality in young infected individuals is likely to be relatively low [2]. We have previously reported that migration did not have a significant impact on our results [9]. Overall, these findings provide convincing evidence that the substantial decrease in HIV prevalence among young people was associated with changes in sexual behaviour.

It is reasonable to believe that asking a woman whether she has ever given birth will yield more reliable answers than asking her whether she has ever had sex, as child-bearing is associated with high respect in this society and is difficult to keep secret. The variable 'ever given birth' was associated with a doubling of the likelihood of HIV infection for single young women. Delays in first pregnancy may be interpreted as strong evidence for the use of an effective preventive strategy, such as abstinence, condom use, or avoidance of marriage to high risk partners. The finding that the proportion who had ever given birth declined among both HIV positive and HIV negative young urban women indicates that decreased fertility among young women was more likely to be due to behavioural change than to the physiological effect of HIV infection. We found that delayed child-bearing seemed to be due to a combination of abstinence, condom use and use of other contraceptives. It is possible that modern contraceptives were mainly used by women engaging in higher risk sexual behaviour in 1999 and that this explains the association with HIV; for as contraceptives rapidly became more common, as well as strongly associated with higher education, this association disappeared.

Fewer women than men reported having had more than one partner during the year prior to each of the three surveys. We also found that fewer men with higher education reported multiple partners in 2003 than in the previous surveys. Before the HIV epidemic in Africa, higher socioeconomic and educational status were associated with more sexual partners [10,11,13]. The finding in the base-line survey that more highly-educated young men and women less frequently reported more than one partner during the past year indicates that the process of change in sexual behaviour started earlier than the mid 1990s. This may be linked to the comprehensive HIV prevention campaigns that were launched in the early 1990s. As people with higher education are usually more concerned with their health, it is likely that they changed their behaviour in response to health-promoting messages [10,12,20].

Consistent condom use has been shown to be effective in protection against HIV transmission, but it also seems that condom use must reach a certain level to influence the HIV epidemic significantly [21,22]. Condom use at last sexual intercourse is often employed as a population-level indicator of frequency, but this has obvious limitations and may not give a representative picture of consistent condom use [23]. 'Condom use ever' is an indicator of acceptability. Both reported condom use 'at last sexual intercourse' and 'ever' increased, especially among urban respondents and people with higher education; these groups were already reporting higher use than rural and less educated respondents in 1995. This suggests that condom promotion has had limited success so far in reaching rural people and those with little education. The Sexual Behaviour Surveys of 2000, 2003 and 2005 also showed lower condom use in rural areas [24-26]. The resistance to condoms may arise from beliefs that they reduce sexual pleasure and male potency, or the belief that condoms are not effective in preventing HIV transmission [24,27,28]. Condom use has also been opposed by many religious communities. Individuals with casual partners during the past year were the only rural group that reported increased condom use; this trend was most evident among those few young women who admitted to this behaviour. A higher frequency of condom use in casual relationships is also reported by other studies from sub-Saharan Africa [29]. Young women with casual partners are probably more conscious of their own risk of becoming HIV infected, as casual sex is considered especially inappropriate for them and is condemned by society.

Frequent dry sex with traditional agents was associated with HIV infection among urban women. It has been believed that this practice increases the susceptibility of women to HIV by creating erosions in the mucous membrane. However, the evidence for increased susceptibility is insubstantial because most studies have, like ours, been cross-sectional, a design that precludes conclusions about causal relationships [30-32]. In any case, according to our results, this practice has become less common, with fewer women in all educational categories reporting it.

The differential HIV trends associated with the education levels of young people indicate that important changes in sexual behaviour have taken place among educated people. If people of different educational levels are part of the same social networks, distinct infection patterns reflect differences in risk exposure provided that the effects of differential mortality and migration are negligible. Thus we believe that most of the changes in reported behaviour observed between 1995 and 2003 are real. However, there are some signs of differential reporting bias in our study. We found that controlling for less risky sexual behaviour substantially reduced the association between HIV and survey time among urban men, but less so among women; this may suggest that self-reports from men about sexual behaviour are more reliable. Studies suggest that respondents, especially women, tend to under-report the number of lifetime sexual partners [33]. Therefore, analyses of associations with, and changes in, self-reported sexual behaviour should be interpreted with caution. This also means that changes in the number of partners during the past year reported by women should be interpreted with extra caution. The finding that more highly educated rural women reported more partners in 2003 than in 1995, whereas all other groups reported fewer, could indicate less reporting bias in this group in 2003. We know that culturally inappropriate sexual behaviour is associated with stigmatization, and social desirability may create reporting bias. Another variable that is known to be associated with social desirability – but also with recall bias – is early sexual experience. Recall bias can be expected to assume greater importance among older age groups. We found indications of under-reporting of sexual experience among both males and females, in that some respondents claiming to be virgins were HIV positive whilst reporting no previous blood transfusions. There is convincing evidence that the dominant form of HIV transmission among adults in sub-Saharan Africa is through heterosexual intercourse [34-38], and other studies have also found HIV infection, STIs and current pregnancy among young people who denied having sex [29,33,37]. Except for individuals who strongly suspected that they were infected, it seems unlikely that respondents' actual HIV status affected the self-reporting in our study, since only 10% of respondents had ever been HIV tested. As the 1995 interview did not include questions about age at sexual debut, we can only compare changes between 1999 and 2003 on this variable, which makes the interpretations more vulnerable to error. We found no visible cohort effect, as could be expected if there had been a real change in age at first sexual intercourse, and the respondents were consistent from survey to survey as to the age at which they reported having had their sexual debut. Instead, we found that among the youngest respondents, fewer admitted having had sex by the age of 15 years in 2003 than in 1999. Analyses of data from the Demographic and Health Surveys, Sexual Behaviour Surveys and from other countries show a similar pattern indicating under-reporting [39-41]. It is probable that as HIV campaigns encouraging delayed sexual debut and abstinence before marriage reach the population, people will report behaviour assumed to be more socially desirable. Other lifetime sexual behaviours such as 'condom use ever' and 'ever given birth' are, however, less prone to recall and social desirability bias, and the reporting of both consistently increased (as might be expected given that the young age cohorts grew older between 1995 and 2003 [see Additional file 17 and 18]). Overall, it seems that bias in our study was mainly related to sexual debut and condom use at last sexual intercourse in 1999. The proportion who said they used a condom at their last sexual intercourse and at their last casual sexual intercourse increased over the 8 year period, but was much lower in 1999 than in 1995. While this is difficult to explain, such inconsistencies in the direction of change may indicate random variation between the surveys, changes in the sample due to migration, changes due to greater absence in 1999, reporting bias, or data entry errors. These inconsistencies are unlikely to be due to misunderstandings of the questions as they were formulated in the same way in all three surveys. However, we observed consistent trends or stability for most indicators, which increases the likelihood that these reflect real behaviour patterns.

The identification of confounding variables for the association between HIV and survey time builds on the assumption that the behaviours reported in 1995 and 2003 reflected the overall level of respondents' risk behaviour. Because we only use rather crude information about behaviour, this model remains quite simplistic. It is not possible in cross-sectional studies to establish whether behaviour change occurred before HIV infection in order for behaviour change to be associated with the HIV infection. Despite these important limitations we believe that since most young HIV infected individuals have been recently infected, the model provides clues to the behaviours that actually contributed to the decline in HIV. The analyses, when not stratifying by education (Tables 4, 5, 6) and when confining analysis to the higher-education groups, identified the same confounders, except that 'frequent dry sex' proved to be the most important for urban women with higher education. Considering the insubstantiality of the evidence for a link between dry sex and HIV infection, we are more inclined to believe that 'any casual partners' and 'ever given birth' were the key factors, as suggested by Table 5.

The data in this study stem from only one urban and one rural community. Generalisation to Zambia as a whole naturally requires further study. However, HIV prevalence levels in these communities are demonstrably comparable to the national urban and rural estimates, and national surveillance data among antenatal attendees have shown declines in prevalence in the 15–24 age-group [10,15,18,42]. Accordingly, these data might well contain important insights regarding overall national patterns of HIV-related behavioural responses.

## Conclusion

We conclude that there is clear evidence of a shift towards safer sexual behaviour among men and women in these communities, especially among young people with higher education. Moreover, these changes are likely to have contributed to the concomitant decline in HIV prevalence observed across the period. However, the results also pinpoint a need for much more effective preventive approaches, targeting rural and less educated people in particular.

## Competing interests

The author(s) declare that they have no competing interests.

## Authors' contributions

IFS analysed and interpreted the data and drafted the manuscript. CM supervised and coordinated the 2003 data collection and took an active part in interpreting the results and revising the manuscript. SS planned and coordinated the 2003 data collection and took an active part in interpreting the results and revising the manuscript. KF made substantial contributions to the conception and design of the surveys, coordinated and supervised the data collection in 1995 and 1999, planned the data collection in 2003, and took an active part in interpreting the results and revising the manuscript. All authors read and approved the final manuscript.

## Pre-publication history

The pre-publication history for this paper can be accessed here:



## Supplementary Material

Additional file 1 Additional table 1. Characteristics of respondents Click here for file

Additional file 2 Additional table 2. Changes in the proportions reporting having any casual partner during the year prior to the survey by marital status, adults aged 15–24, 1995–2003 Click here for file

Additional file 3 Additional table 3. Changes in the proportions reporting condom use at last sexual intercourse by educational attainment among adults aged 15–24, 1995–2003 Click here for file

Additional file 4 Additional table 4. Changes in the proportions of HIV-negative women aged 15–24 who had ever given birth by educational attainment, 1995–2003 Click here for file

Additional file 5 Additional table 5. HIV prevalence by whether 'ever given birth', stratified by marital status. Women aged 15–24. Click here for file

Additional file 6 Additional table 6. Age-adjusted odds ratio (AOR) of having given birth comparing 2003 and 1995 (2003 and 1999 for modern contraceptives) and adjusting for 'current use of modern contraceptives', 'used condom at last sexual intercourse' and abstinence among rural and urban women aged 15–24 Click here for file

Additional file 7 Additional table 7. Changes in the proportion of all women aged 15–24 who frequently use traditional agents before sex to make the vagina drier, by educational attainment, 1995–2003 Click here for file

Additional file 8 Additional table 8. Proportions of all women aged 15–24 who frequently use traditional agents before sex to make the vagina drier, by educational attainment, 1995–2003 Click here for file

Additional file 9 Additional table 9. Changes in the proportions of females aged 15–24 reporting current use of modern contraceptives by educational attainment, 1995–2003 Click here for file

Additional file 10 Additional table 10. Changes in the proportions reporting current use of modern contraceptives by educational attainment among females aged 15–24, 1995–2003 Click here for file

Additional file 11 Additional table 11. Changes in the proportions reporting ever having sex by educational attainment among adults aged 15–24, 1995–2003 Click here for file

Additional file 12 Additional table 12. Changes in the median reported age at sexual debut by educational attainment among adults aged 15–24, 1995–2003 Click here for file

Additional file 13 Additional figure 1. Percentages reporting having had sex by the age of 15 in 1999 and in 2003 based on the age groups of the respondents in 1999 Click here for file

Additional file 14 Additional table 13. Percentages reporting having had sex by the age of 15 in 1999 and in 2003 based on the age groups of the respondents in 1999. Click here for file

Additional file 15 Additional table 14. HIV prevalence among young people aged 15–24 reporting 'ever' and 'never' sexual activity Click here for file

Additional file 16 Additional table 15. Age-adjusted odds ratio (AOR) of risk of HIV infection by survey time comparing 2003 and 1995, adjusting for sexual behaviour variables, for urban and rural men and women aged 15–24 with higher education (≥10 years of schooling) Click here for file

Additional file 17 Additional figure 2. Proportions who reported ever having used a condom in 1995, 1999 and 2003 based on the age groups of the respondents in 1995. The denominator is the number of sexually active respondents Click here for file

Additional file 18 Additional figure 3. Proportion of women reporting 'ever given birth' in 1995, 1999 and 2003 based on the age groups in 1995 Click here for file
