# Supplementary material for: Associations between sexual behaviour change in young people and decline in HIV prevalence in Zambia
Source: BMC Public Health. 2007 Apr 23;7:60. doi: 10.1186/1471-2458-7-60 (PMC1868719; doi:10.1186/1471-2458-7-60)
Supplement: Additional file 1 — Additional table 1. Characteristics of respondents [file 1471-2458-7-60-S1.doc]

**Characteristics of respondents**

| **Age 15-24** | | | | | | | | | | | | | |
| --- | --- | --- | --- | --- | --- | --- | --- | --- | --- | --- | --- | --- | --- |
|  | | **Rural** | | | | | | **Urban** | | | | | |
|  | | **1995** | | **1999** | | **2003** | | **1995** | | **1999** | | **2003** | |
|  | |  | N |  | N |  | N |  | N |  | N |  | N |
| Sex distribution (%) | M | 43.2 | 197 | 40.5 | 290 | 44.9 | 432 | 37.9 | 479 | 40.8 | 1230 | 42.1 | 1675 |
|  | F | 56.8 | 259 | 59.5 | 426 | 55.1 | 530 | 62.1 | 1264 | 59.2 | 1230 | 57.9 | 1675 |
| Mean age in years (95% CI) | M | 19.9 (19.5-20.2) | 197 | 19.6 (19.3-20.0) | 290 | 19.7 (19.4-20.0) | 432 | 18.9 (18.7-19.2) | 479 | 19.3 (19.1-19.6) | 502 | 19.5 (19.3-19.7) | 705 |
|  | F | 19.3 (18.9-19.6) | 259 | 19.2 (18.9-19.4) | 426 | 19.4 (19.2-19.7) | 530 | 19.1 (18.9-19.3) | 785 | 19.4 (19.2-19.6) | 728 | 19.5 (19.3-19.6) | 970 |
| Median age in years (IQR) | M | 20 (18-22) | 197 | 20 (17-22) | 290 | 20 (17.25-22) | 432 | 19 (17-21) | 479 | 19 (17-22) | 502 | 19 (17-22) | 705 |
|  | F | 19 (17-22) | 259 | 19 (17-22) | 426 | 20 (17-22) | 530 | 19 (17-22) | 785 | 19 (17-22) | 728 | 19 (17-22) | 970 |
| Mean number of school years (95% CI) | M | 7.0 (6.6-7.5) | 195 | 6.4 (6.0-6.7) | 290 | 7.1 (6.7-7.5) | 326 | 9.2 (9.0-9.4) | 477 | 10.1 (9.9-10.3) | 496 | 10.6 (10.4-10.8) | 696 |
|  | F | 6.3 (5.9-6.6) | 256 | 5.4 (5.2-5.7) | 425 | 5.7 (5.4-6.0) | 485 | 8.4 (8.2-8.6) | 778 | 9.2 (9.0-9.4) | 725 | 9.9 (9.8-10.1) | 957 |
| Median number of school years (IQR) | M | 7 (6-9) | 195 | 7 (4-8) | 290 | 7 (5-9) | 326 | 9 (7-11) | 477 | 10 (9-12) | 496 | 11 (9-12) | 696 |
|  | F | 7 (5-8) | 256 | 6 (4-7) | 425 | 6 (4-8) | 485 | 9 (7-10) | 778 | 9 (7-12) | 725 | 11 (8-12) | 957 |
