# Supplementary material for: Associations between sexual behaviour change in young people and decline in HIV prevalence in Zambia
Source: BMC Public Health. 2007 Apr 23;7:60. doi: 10.1186/1471-2458-7-60 (PMC1868719; doi:10.1186/1471-2458-7-60)
Supplement: Additional file 2 — Additional table 2. Changes in the proportions reporting having any casual partner during the year prior to the survey by marital status, adults aged 15–24, 1995–2003 [file 1471-2458-7-60-S2.doc]

**Changes in the proportions reporting having any casual partner during the year prior to the survey by marital status, adults aged 15-24, 1995-2003**

|  |  |  | **Single** | | | | | | **Married** | | | | | |
| --- | --- | --- | --- | --- | --- | --- | --- | --- | --- | --- | --- | --- | --- | --- |
| **Residence** |  | **Year** | **%** | **N** | **Crude OR** | **95% CI** | **AOR** | **95%**  **CI** | **%** | **N** | **Crude OR** | **95% CI** | **AOR** | **95%**  **CI** |
| **Rural** | **Males** | *1995* | 50 | 106 | Ref. |  | Ref. |  | 34 | 35 | Ref. |  | Ref. |  |
|  |  | *1999* | 60 | 139 | 1.48 | 0.64-3.42 | 1.49 | 0.67-3.32 | 39 | 61 | 1.24 | 0.63-2.43 | 1.21 | 0.64-2.30 |
|  |  | *2003* | 55 | 126 | 1.21 | 0.66-2.23 | 1.21 | 0.66-2.22 | 35 | 62 | 1.05 | 0.47-2.39 | 1.09 | 0.49-2.45 |
|  | **Females** | *1995* | 25 | 51 | Ref. |  | Ref. |  | 6 | 126 | Ref. |  | Ref. |  |
|  |  | *1999* | 32 | 74 | 1.40 | 0.77-2.56 | 1.21 | 0.66-2.22 | 4 | 245 | 0.69 | 0.32-1.52 | 0.63 | 0.28-1.42 |
|  |  | *2003* | 14 | 66 | **0.46** | **0.25-0.86** | **0.41** | **0.24-0.71** | 5 | 262 | 0.71 | 0.30-1.69 | 0.73 | 0.30-1.80 |
| **Urban** | **Males** | *1995* | 46 | 240 | Ref. |  | Ref. |  | 50 | 14 | Ref. |  | Ref. |  |
|  |  | *1999* | 45 | 195 | 0.95 | 0.66-1.37 | 0.94 | 0.66-1.36 | 64 | 11 | 1.75 | 0.41-7.47 | 1.73 | 0.42-7.04 |
|  |  | *2003* | 42 | 229 | 0.87 | 0.63-1.21 | 0.86 | 0.63-1.16 | 38 | 13 | 0.62 | 0.13-3.05 | 0.63 | 0.13-3.01 |
|  | **Females** | *1995* | 17 | 235 | Ref. |  | Ref. |  | 4 | 169 | Ref. |  | Ref. |  |
|  |  | *1999* | 24 | 218 | 1.52 | 0.93-2.48 | 1.50 | 0.90-2.49 | 3 | 104 | 0.81 | 0.14-4.59 | 0.78 | 0.14-4.41 |
|  |  | *2003* | 15 | 221 | 0.86 | 0.54-1.36 | 0.83 | 0.51-1.34 | 2 | 130 | 0.64 | 0.37-1.12 | 0.58 | 0.32-1.07 |
