# Supplementary material for: Associations between sexual behaviour change in young people and decline in HIV prevalence in Zambia
Source: BMC Public Health. 2007 Apr 23;7:60. doi: 10.1186/1471-2458-7-60 (PMC1868719; doi:10.1186/1471-2458-7-60)
Supplement: Additional file 3 — Additional table 3. Changes in the proportions reporting condom use at last sexual intercourse by educational attainment among adults aged 15–24, 1995–2003 [file 1471-2458-7-60-S3.doc]

**Changes in the proportions reporting condom use at last sexual intercourse by educational attainment among adults aged 15-24, 1995-2003**

|  | **School years** |  | **0-7** | | | | | | **8-9** | | | | | | **10+** | | | | | |
| --- | --- | --- | --- | --- | --- | --- | --- | --- | --- | --- | --- | --- | --- | --- | --- | --- | --- | --- | --- | --- |
| **Residence** |  | **Year** | **%** | **N** | **Crude OR** | **95% CI** | **AOR** | **95%**  **CI** | **%** | **N** | **Crude OR** | **95% CI** | **AOR** | **95%**  **CI** | **%** | **N** | **Crude OR** | **95% CI** | **AOR** | **95%**  **CI** |
| **Rural** | **Males** | *1995* | 23 | 83 | Ref. |  | Ref. |  | 58 | 33 | Ref. |  | Ref. |  | 63 | 19 | Ref. |  | Ref. |  |
|  |  | *1999* | 28 | 134 | 1.28 | 0.72-2.31 | 1.18 | 0.65-2.13 | 23 | 31 | **0.21** | **0.06-0.80** | **0.21** | **0.06-0.79** | 19 | 16 | **0.13** | **0.06-0.32** | **0.13** | **0.05-0.31** |
|  |  | *2003* | 18 | 74 | 0.72 | 0.34-1.52 | 0.72 | 0.34-1.55 | 52 | 27 | 0.79 | 0.21-3.07 | 0.77 | 0.18-3.27 | 62 | 32 | 0.97 | 0.50-1.90 | 0.99 | 0.55-1.80 |
|  | **Females** | *1995* | 9 | 116 | Ref. |  | Ref. |  | 21 | 33 | Ref. |  | Ref. |  | 22 | 9 | Ref. |  | Ref. |  |
|  |  | *1999* | 20 | 223 | 2.35 | 0.89-6.21 | 2.28 | 0.85-6.06 | 16 | 38 | 0.70 | 0.15-3.21 | 0.64 | 0.12-3.36 | 14 | 7 | 0.58 | 0.02-14.1 | 0.34 | 0.01-10.3 |
|  |  | *2003* | 13 | 189 | 1.39 | 0.59-3.28 | 1.41 | 0.60-3.29 | 26 | 39 | 1.28 | 0.49-3.31 | 1.27 | 0.46-3.49 | 70 | 20 | **8.17** | **3.25-20.5** | **11.3** | **2.11-60.8** |
| **Urban** | **Males** | *1995* | 42 | 59 | Ref. |  | Ref. |  | 47 | 85 | Ref. |  | Ref. |  | 63 | 103 | Ref. |  | Ref. |  |
|  |  | *1999* | 13 | 30 | **0.21** | **0.07-0.59** | **0.21** | **0.08-0.57** | 29 | 70 | 0.45 | 0.20-1.02 | 0.51 | 0.22-1.19 | 27 | 150 | **0.21** | **0.12-0.37** | **0.22** | **0.12-0.37** |
|  |  | *2003* | 51 | 35 | 1.44 | 0.45-4.60 | 1.40 | 0.48-4.08 | 49 | 45 | 1.08 | 0.64-1.81 | 0.85 | 0.50-1.46 | 75 | 169 | 1.71 | 0.95-3.08 | 1.65 | 0.91-3.00 |
|  | **Females** | *1995* | 30 | 145 | Ref. |  | Ref. |  | 35 | 140 | Ref. |  | Ref. |  | 42 | 124 | Ref. |  | Ref. |  |
|  |  | *1999* | 23 | 126 | 0.69 | 0.38-1.25 | 0.63 | 0.33-1.19 | 19 | 108 | **0.45** | **0.24-0.85** | **0.45** | **0.23-0.88** | 29 | 190 | **0.58** | **0.36-0.93** | **0.47** | **0.28-0.80** |
|  |  | *2003* | 38 | 73 | 1.43 | 0.64-3.18 | 1.58 | 0.74-3.37 | 55 | 73 | 2.25 | 1.48-3.43 | **2.16** | **1.48-3.14** | 64 | 199 | **2.44** | **1.31-4.54** | **2.40** | **1.24-4.62** |
