# Supplementary material for: Associations between sexual behaviour change in young people and decline in HIV prevalence in Zambia
Source: BMC Public Health. 2007 Apr 23;7:60. doi: 10.1186/1471-2458-7-60 (PMC1868719; doi:10.1186/1471-2458-7-60)
Supplement: Additional file 4 — Additional table 4. Changes in the proportions of HIV-negative women aged 15–24 who had ever given birth by educational attainment, 1995–2003 [file 1471-2458-7-60-S4.doc]

**Changes in the proportions of HIV-negative women aged 15-24 who had ever given birth by educational attainment, 1995-2003**

|  | **School years** |  | **0-7** | | | | | | **8-9** | | | | | | **10+** | | | | | |
| --- | --- | --- | --- | --- | --- | --- | --- | --- | --- | --- | --- | --- | --- | --- | --- | --- | --- | --- | --- | --- |
| **Residence** |  | **Year** | **%** | **N** | **Crude OR** | **95% CI** | **AOR** | **95%**  **CI** | **%** | **N** | **Crude OR** | **95% CI** | **AOR** | **95%**  **CI** | **%** | **N** | **Crude OR** | **95% CI** | **AOR** | **95%**  **CI** |
| **Rural** | **Females** | *1995* | 53 | 148 | Ref. |  | Ref. |  | 46 | 35 | Ref. |  | Ref. |  | 70 | 10 | Ref. |  | Ref. |  |
|  |  | *1999* | 60 | 275 | 1.37 | 0.58-3.24 | 1.67 | 0.67-4.13 | 49 | 45 | 1.14 | 0.47-2.73 | 1.19 | 0.43-3.28 | 33 | 12 | 0.21 | 0.04-1.06 | 0.67 | 0.17-2.70 |
|  |  | *2003* | 67 | 299 | **1.81** | **1.02-3.21** | **1.89** | **1.10-3.25** | 44 | 71 | 0.92 | 0.42-2.00 | 0.79 | 0.24-2.62 | 22 | 40 | **0.12** | **0.06-0.24** | **0.17** | **0.09-0.34** |
| **Urban** | **Females** | *1995* | 31 | 211 | Ref. |  | Ref. |  | 36 | 179 | Ref. |  | Ref. |  | 28 | 142 | Ref. |  | Ref. |  |
|  |  | *1999* | 32 | 139 | 1.04 | 0.56-1.92 | 0.73 | 0.41-1.28 | 28 | 142 | 0.70 | 0.35-1.42 | **0.39** | **0.17-0.90** | 16 | 235 | **0.48** | **0.30-0.75** | 0.53 | 0.28-1.01 |
|  |  | *2003* | 26 | 118 | 0.80 | 0.33-1.93 | 0.50 | 0.19-1.30 | 29 | 121 | 0.73 | 0.49-1.09 | **0.36** | **0.16-0.83** | 19 | 397 | **0.61** | **0.38-0.99** | **0.56** | **0.33-0.96** |
