# Supplementary material for: Associations between sexual behaviour change in young people and decline in HIV prevalence in Zambia
Source: BMC Public Health. 2007 Apr 23;7:60. doi: 10.1186/1471-2458-7-60 (PMC1868719; doi:10.1186/1471-2458-7-60)
Supplement: Additional file 5 — Additional table 5. HIV prevalence by whether 'ever given birth', stratified by marital status. Women aged 15–24. [file 1471-2458-7-60-S5.doc]

**HIV prevalence by whether ‘ever given birth’, stratified by marital status. Women aged 15-24.**

|  |  | **Single** | | | | | | | | | | | | **Married** | | | | | | | | | | | |
| --- | --- | --- | --- | --- | --- | --- | --- | --- | --- | --- | --- | --- | --- | --- | --- | --- | --- | --- | --- | --- | --- | --- | --- | --- | --- |
|  |  | **Rural** | | | | | | **Urban** | | | | | | **Rural** | | | | | | **Urban** | | | | | |
|  |  | **%** | **N** | **Crude OR** | **95% CI** | **AOR** | **95% CI** | **%** | **N** | **Crude OR** | **95% CI** | **AOR** | **95% CI** | **%** | **N** | **Crude OR** | **95% CI** | **AOR** | **95% CI** | **%** | **N** | **Crude OR** | **95% CI** | **AOR** | **95% CI** |
| ***1995*** | ***No*** | 8.0 | 87 | Ref. |  | Ref. |  | 11.8 | 397 | Ref. |  | Ref. |  | 22.2 | 18 | Ref. |  | Ref. |  | 23.8 | 21 | Ref. |  | Ref. |  |
|  | ***Yes*** | 20.0 | 20 | 2.86 | 0.91-8.98 | 1.24 | 0.37-4.19 | 29.4 | 102 | **3.10** | **1.74-5.52** | **2.20** | **1.43-3.37** | 20.2 | 99 | 0.89 | 0.34-2.32 | 0.60 | 0.22-1.63 | 41.3 | 138 | 2.25 | 0.62-8.20 | 1.64 | 0.34-7.85 |
| ***1999*** | ***No*** | 5.0 | 100 | Ref. |  | Ref. |  | 13.6 | 441 | Ref. |  | Ref. |  | 4.3 | 47 | Ref. |  | Ref. |  | 23.5 | 17 | Ref. |  | Ref. |  |
|  | ***Yes*** | 13.0 | 23 | **2.85** | **1.37-5.93** | 1.07 | 0.17-6.66 | 28.0 | 75 | **2.47** | **1.59-3.84** | **1.68** | **1.05-2.71** | 11.9 | 176 | 3.05 | 0.68-13.6 | 2.02 | 0.48-8.55 | 26.9 | 78 | 1.20 | 0.25-5.79 | 0.60 | 0.18-2.00 |
| ***2003*** | ***No*** | 0.7 | 139 | Ref. |  | Ref. |  | 9.4 | 524 | Ref. |  | Ref. |  | 3.1 | 32 | Ref. |  | Ref. |  | 24.0 | 25 | Ref. |  | Ref. |  |
|  | ***Yes*** | 17.6 | 34 | **29.6** | **3.53-248** | **17.2** | **1.62-183** | 18.0 | 78 | **2.12** | **1.13-3.97** | 1.71 | 0.92-3.20 | 8.3 | 217 | 2.80 | 0.68-11.6 | 2.23 | 0.54-9.21 | 22.6 | 93 | 0.92 | 0.38-2.22 | 0.90 | 0.37-2.17 |
