# Supplementary material for: Associations between sexual behaviour change in young people and decline in HIV prevalence in Zambia
Source: BMC Public Health. 2007 Apr 23;7:60. doi: 10.1186/1471-2458-7-60 (PMC1868719; doi:10.1186/1471-2458-7-60)
Supplement: Additional file 6 — Additional table 6. Age-adjusted odds ratio (AOR) of having given birth comparing 2003 and 1995 (2003 and 1999 for modern contraceptives) and adjusting for 'current use of modern contraceptives', 'used condom at last sexual intercourse' and abstinence among rural and urban women aged 15–24 [file 1471-2458-7-60-S6.doc]

**Age-adjusted odds ratio (AOR) of having given birth comparing 2003 and 1995 (2003 and 1999 for modern contraceptives) and adjusting for ‘current use of modern contraceptives’, ‘used condom at last sexual intercourse’ and abstinence among rural and urban women aged 15-24**

**a) Urban women**

| **One by one behaviour indicator included** | | | | | |
| --- | --- | --- | --- | --- | --- |
| **Beh. Indicator** | | **Chi square** | **Chi-square change** | **AOR** | **95% CI** |
| Comparing 2003 and 1995 | Condom use at last sexual intercourse | **109.69** | **18.86** | **0.46** | **0.27-0.76** |
|  | Ever used condom | **126.72** | **35.89** | **0.54** | **0.30-0.96** |
|  | Sexually active last year | **36.19** | **-54.64** | **0.51** | **0.32-0.84** |
|  | |  |  |  |  |
| Comparing 2003 and 1999 | Modern contraceptive use | **112.12** | **21.29** | **1.02** | **0.72-1.44** |

Notes: **Before adding behaviour variables Chi square was 90.83 and AOR for having given birth 0.33 (0.20-0.55) in 2003 compared to 1995 and 0.77 (0.53-1.11) in 2003 compared to 1999**. If chi square >|3.84|, the added variable is a confounding variable. The denominator is the total number of women.

b)Rural women

|  | | **One by one behaviour indicator included** | | | |
| --- | --- | --- | --- | --- | --- |
| **Beh. Indicator** | | **Chi square** | **Chi-square change** | **AOR** | **95% CI** |
| Comparing 2003 and 1995 | Condom use at last sexual intercourse* | 40.51 | -60.0 | 1.81 | 1.10-2.97 |
|  | Ever used condom* | 74.83 | -25.68 | 2.14 | 1.30-3.51 |
|  | Sexually active last year* | 50.52 | -49.99 | 1.71 | 0.99-2.94 |
|  | |  |  |  |  |
| Comparing 2003 and 1999 | Modern contraceptive use | **105.87** | **5.36** | **0.98** | **0.53-1.79** |
|  | |  |  |  |  |
|  | |  |  |  |  |

Notes: **Before adding behaviour variables Chi square was 100.51 and AOR for having given birth 1.35 (0.78-2.33) in 2003 compared to 1995 and 0.84 (0.44-1.60) in 2003 compared to 1999.** If chi square changed >|3.84|, the added variable is a confounding variable. The denominator is the total number of women. *The Chi square change is significant, but the AOR is further from 1, which means that adjusting for ‘condom use at last sexual intercourse’, ‘ever condom use’ or sexual activity last year increases the strength of the association between ‘ever given birth’ and survey time, rather than reducing it.
