# Supplementary material for: Associations between sexual behaviour change in young people and decline in HIV prevalence in Zambia
Source: BMC Public Health. 2007 Apr 23;7:60. doi: 10.1186/1471-2458-7-60 (PMC1868719; doi:10.1186/1471-2458-7-60)
Supplement: Additional file 7 — Additional table 7. Changes in the proportion of all women aged 15–24 who frequently use traditional agents before sex to make the vagina drier, by educational attainment, 1995–2003 [file 1471-2458-7-60-S7.doc]

**Changes in the proportion of all women aged 15-24 who frequently use traditional agents before sex to make the vagina drier, by educational attainment, 1995-2003**

|  | **School years** |  | **0-7** | | | | | | **8-9** | | | | | | **10+** | | | | | |
| --- | --- | --- | --- | --- | --- | --- | --- | --- | --- | --- | --- | --- | --- | --- | --- | --- | --- | --- | --- | --- |
| **Residence** |  | **Year** | **%** | **N** | **Crude OR** | **95% CI** | **AOR** | **95%**  **CI** | **%** | **N** | **Crude OR** | **95% CI** | **AOR** | **95%**  **CI** | **%** | **N** | **Crude OR** | **95% CI** | **AOR** | **95%**  **CI** |
| **Rural** | **Females** | *1995* | 41 | 133 | Ref. |  | Ref. |  | 28 | 39 | Ref. |  | Ref. |  | 13 | 15 | Ref. |  | Ref. |  |
|  |  | *1999* | 21 | 312 | **0.39** | **0.26-0.60** | **0.42** | **0.27-0.63** | 15 | 55 | 0.43 | 0.12-1.54 | 0.48 | 0.12-1.85 | 7 | 14 | 0.50 | 0.03-9.89 | 0.30 | 0.01-17.4 |
|  |  | *2003* | 19 | 291 | **0.33** | **0.18-0.62** | **0.32** | **0.17-0.60** | 4 | 47 | **0.11** | **0.01-0.96** | **0.11** | **0.01-0.87** | 4 | 26 | 0.26 | 0.02-3.40 | 0.22 | 0.01-4.61 |
| **Urban** | **Females** | *1995* | 22 | 163 | Ref. |  | Ref. |  | 20 | 134 | Ref. |  | Ref. |  | 17 | 128 | Ref. |  | Ref. |  |
|  |  | *1999* | 4 | 160 | **0.14** | **0.03-0.59** | **0.15** | **0.03-0.66** | 4 | 159 | **0.16** | **0.05-0.49** | **0.17** | **0.05-0.55** | 0.4 | 271 | **0.02** | **0.003-0.11** | **0.03** | **0.01-0.13** |
|  |  | *2003* | 12 | 96 | 0.50 | 0.17-1.48 | 0.47 | 0.17-1.27 | 7 | 91 | **0.28** | **0.10-0.81** | **0.23** | **0.08-0.69** | 2 | 239 | **0.10** | **0.03-0.30** | **0.11** | **0.04-0.32** |
