# Supplementary material for: Associations between sexual behaviour change in young people and decline in HIV prevalence in Zambia
Source: BMC Public Health. 2007 Apr 23;7:60. doi: 10.1186/1471-2458-7-60 (PMC1868719; doi:10.1186/1471-2458-7-60)
Supplement: Additional file 8 — Additional table 8. Proportions of all women aged 15–24 who frequently use traditional agents before sex to make the vagina drier, by educational attainment, 1995–2003 [file 1471-2458-7-60-S8.doc]

**Proportions of all women aged 15-24 who frequently use traditional agents before sex to make the vagina drier, by educational attainment, 1995-2003**

|  | **Year** |  | **1995** | | | | | | **1999** | | | | | | | **2003** | | | | |
| --- | --- | --- | --- | --- | --- | --- | --- | --- | --- | --- | --- | --- | --- | --- | --- | --- | --- | --- | --- | --- |
| **Residence** |  | **School years** | **%** | **N** | **Crude OR** | **95% CI** | **AOR** | **95%**  **CI** | **%** | **N** | **Crude OR** | **95% CI** | **AOR** | **95%**  **CI** | **%** | **N** | **Crude OR** | **95% CI** | **AOR** | **95%**  **CI** |
| **Rural** | **Females** | 0-7 | 41 | 133 | Ref. |  | Ref. |  | 21 | 312 | Ref. |  | Ref. |  | 19 | 291 | Ref. |  | Ref. |  |
|  |  | *8-9* | 28 | 39 | 0.57 | 0.27-1.21 | 0.57 | 0.26-1.22 | 15 | 55 | 0.63 | 0.17-2.41 | 0.60 | 0.17-2.09 | 4 | 47 | 0.20 | 0.02-1.63 | 0.18 | 0.02-1.34 |
|  |  | *10+* | 13 | 15 | 0.23 | 0.04-1.32 | 0.21 | 0.03-1.34 | 7 | 14 | **0.29** | **0.09-0.90** | 0.29 | 0.08-1.05 | 4 | 26 | **0.18** | **0.08-0.40** | **0.15** | **0.06-0.37** |
| **Urban** | **Females** | 0-7 | 22 | 163 | Ref. |  | Ref. |  | 4 | 160 | Ref. |  | Ref. |  | 12 | 96 | Ref. |  | Ref. |  |
|  |  | *8-9* | 20 | 134 | 0.89 | 0.55-1.45 | 0.85 | 0.50-1.46 | 4 | 159 | 1.01 | 0.43-2.36 | 0.86 | 0.37-1.99 | 7 | 91 | **0.49** | **0.28-0.87** | **0.44** | **0.21-0.91** |
|  |  | *10+* | 17 | 128 | 0.73 | 0.35-1.54 | 0.57 | 0.27-1.18 | 0.4 | 271 | **0.10** | **0.01-0.96** | **0.07** | **0.01-0.69** | 2 | 239 | **0.15** | **0.06-0.39** | **0.12** | **0.05-0.28** |
