# Supplementary material for: Associations between sexual behaviour change in young people and decline in HIV prevalence in Zambia
Source: BMC Public Health. 2007 Apr 23;7:60. doi: 10.1186/1471-2458-7-60 (PMC1868719; doi:10.1186/1471-2458-7-60)
Supplement: Additional file 9 — Additional table 9. Changes in the proportions of females aged 15–24 reporting current use of modern contraceptives by educational attainment, 1995–2003 [file 1471-2458-7-60-S9.doc]

**Changes in the proportions of females aged 15-24 reporting current use of modern contraceptives by educational attainment, 1995-2003**

|  | **School years** | | **0-7** | | | | | | **8-9** | | | | | | | **10+** | | | | |
| --- | --- | --- | --- | --- | --- | --- | --- | --- | --- | --- | --- | --- | --- | --- | --- | --- | --- | --- | --- | --- |
| **Residence** |  | **Year** | **%** | **N** | **Crude OR** | **95% CI** | **AOR** | **95%**  **CI** | **%** | **N** | **Crude OR** | **95% CI** | **AOR** | **95%**  **CI** | **%** | **N** | **Crude OR** | **95% CI** | **AOR** | **95%**  **CI** |
| **Rural** | **Females** | *1999* | 15 | 324 | Ref. |  | Ref. |  | 27 | 56 | Ref. |  | Ref. |  | 20 | 15 | Ref. |  | Ref. |  |
|  |  | *2003* | 13 | 309 | 0.88 | 0.55-1.39 | 0.82 | 0.53-1.26 | 19 | 57 | 0.65 | 0.36-1.20 | 0.66 | 0.34-1.27 | 50 | 34 | **4.00** | **2.15-7.43** | **3.13** | **1.51-6.49** |
| **Urban** | **Females** | *1999* | 19 | 167 | Ref. |  | Ref. |  | 28 | 169 | Ref. |  | Ref. |  | 31 | 280 | Ref. |  | Ref. |  |
|  |  | *2003* | 35 | 110 | 2.23 | 0.92-5.40 | 1.86 | 0.84-4.14 | 32 | 102 | 1.24 | 0.78-1.97 | 0.90 | 0.58-1.38 | 40 | 280 | **1.53** | **1.10-2.12** | 1.26 | 0.95-1.69 |
