# Supplementary material for: Associations between sexual behaviour change in young people and decline in HIV prevalence in Zambia
Source: BMC Public Health. 2007 Apr 23;7:60. doi: 10.1186/1471-2458-7-60 (PMC1868719; doi:10.1186/1471-2458-7-60)
Supplement: Additional file 10 — Additional table 10. Changes in the proportions reporting current use of modern contraceptives by educational attainment among females aged 15–24, 1995–2003 [file 1471-2458-7-60-S10.doc]

**Changes in the proportions reporting current use of modern contraceptives by educational attainment among females aged 15-24, 1995-2003**

| **Year** |  | **1999** | | | | | | **2003** | | | | | |
| --- | --- | --- | --- | --- | --- | --- | --- | --- | --- | --- | --- | --- | --- |
| **Residence** | **School years** | **%** | **N** | **Crude OR** | **95% CI** | **AOR** | **95%**  **CI** | **%** | **N** | **Crude OR** | **95% CI** | **AOR** | **95%**  **CI** |
| **Rural** | *0-7* | 15 | 324 | Ref. |  | Ref. |  | 13 | 309 | Ref. |  | Ref. |  |
|  | *8-9* | 27 | 56 | **2.16** | **1.51-3.07** | **2.09** | **1.38-3.17** | 19 | 57 | 1.61 | 0.90-2.86 | 1.68 | 0.97-2.92 |
|  | *10+* | 20 | 15 | 1.47 | 0.78-2.78 | 1.62 | 0.84-3.15 | 50 | 34 | **6.72** | **4.52-10.0** | **6.73** | **4.96-9.14** |
| **Urban** | *0-7* | 19 | 167 | Ref. |  | Ref. |  | 35 | 110 | Ref. |  | Ref. |  |
|  | *8-9* | 28 | 169 | **1.63** | **1.21-2.18** | **1.55** | **1.01-2.38** | 32 | 102 | 0.91 | 0.42-1.97 | 0.81 | 0.37-1.75 |
|  | *10+* | 31 | 280 | **1.87** | **1.20-2.92** | 1.55 | 0.98-2.45 | 40 | 280 | 1.28 | 0.62-2.63 | 1.05 | 0.58-1.89 |
