# Supplementary material for: Associations between sexual behaviour change in young people and decline in HIV prevalence in Zambia
Source: BMC Public Health. 2007 Apr 23;7:60. doi: 10.1186/1471-2458-7-60 (PMC1868719; doi:10.1186/1471-2458-7-60)
Supplement: Additional file 11 — Additional table 11. Changes in the proportions reporting ever having sex by educational attainment among adults aged 15–24, 1995–2003 [file 1471-2458-7-60-S11.doc]

**Changes in the proportions reporting ever having sex by educational attainment among adults aged 15-24, 1995-2003**

|  | **School years** |  | **0-7** | | | | | | **8-9** | | | | | | **10+** | | | | | |
| --- | --- | --- | --- | --- | --- | --- | --- | --- | --- | --- | --- | --- | --- | --- | --- | --- | --- | --- | --- | --- |
| **Residence** |  | **Year** | **%** | **N** | **Crude OR** | **95% CI** | **AOR** | **95%**  **CI** | **%** | **N** | **Crude OR** | **95% CI** | **AOR** | **95%**  **CI** | **%** | **N** | **Crude OR** | **95% CI** | **AOR** | **95%**  **CI** |
| **Rural** | **Males** | *1999* | 90 | 202 | Ref. |  | Ref. |  | 88 | 59 | Ref. |  | Ref. |  | 97 | 29 | Ref. |  | Ref. |  |
|  |  | *2003* | 79 | 196 | **0.43** | **0.20-0.89** | **0.42** | **0.18-0.96** | 78 | 58 | **0.47** | **0.25-0.87** | **0.25** | **0.07-0.86** | 72 | 72 | 0.09 | 0.01-1.05 | **0.08** | **0.01-0.75** |
|  | **Females** | *1999* | 87 | 342 | Ref. |  | Ref. |  | 81 | 64 | Ref. |  | Ref. |  | 78 | 18 | Ref. |  | Ref. |  |
|  |  | *2003* | 84 | 356 | 0.79 | 0.47-1.34 | 0.65 | 0.35-1.22 | 69 | 81 | **0.52** | **0.40-0.67** | **0.58** | **0.38-0.88** | 67 | 48 | 0.57 | 0.28-1.15 | 0.33 | 0.09-1.18 |
| **Urban** | **Males** | *1999* | 60 | 68 | Ref. |  | Ref. |  | 57 | 141 | Ref. |  | Ref. |  | 70 | 287 | Ref. |  | Ref. |  |
|  |  | *2003* | 71 | 73 | 1.63 | 0.97-2.74 | 0.96 | 0.42-2.23 | 50 | 143 | 0.73 | 0.33-1.64 | 0.60 | 0.30-1.18 | 62 | 477 | **0.69** | **0.53-0.89** | 0.74 | 0.52-1.06 |
|  | **Females** | *1999* | 66 | 193 | Ref. |  | Ref. |  | 65 | 206 | Ref. |  | Ref. |  | 65 | 324 | Ref. |  | Ref. |  |
|  |  | *2003* | 59 | 193 | 0.75 | 0.46-1.22 | 0.70 | 0.44-1.09 | 55 | 198 | 0.66 | 0.34-1.26 | 0.70 | 0.31-1.58 | 54 | 564 | **0.61** | **0.47-0.80** | **0.51** | **0.38-0.69** |
