# Supplementary material for: Associations between sexual behaviour change in young people and decline in HIV prevalence in Zambia
Source: BMC Public Health. 2007 Apr 23;7:60. doi: 10.1186/1471-2458-7-60 (PMC1868719; doi:10.1186/1471-2458-7-60)
Supplement: Additional file 12 — Additional table 12. Changes in the median reported age at sexual debut by educational attainment among adults aged 15–24, 1995–2003 [file 1471-2458-7-60-S12.doc]

**Changes in the median reported age at sexual debut by educational attainment among adults aged 15-24, 1995-2003**

|  | **School years** |  | **0-7** | | | | | **8-9** | | | | | **10+** | | | |
| --- | --- | --- | --- | --- | --- | --- | --- | --- | --- | --- | --- | --- | --- | --- | --- | --- |
| **Residence** |  | **Year** | **Median** | **IQR** | **N** | **p-value** | | **Median** | **IQR** | **N** | **p-value** | | **Median** | **IQR** | **N** | **p-value** |
| **Rural** | **Males** | *1999* | 15 | 14-17 | 197 | 0.03 | | 15 | 14-18 | 57 | **0.013** | | 16 | 15-17 | 29 | **0.005** |
|  |  | *2003* | 16 | 15-16 | 195 |  |  | 17 | 15-18 | 58 |  |  | 18 | 16-21 | 72 |  |
|  |  |  |  |  |  |  |  |  |  |  |  |  |  |  |  |  |
|  | **Females** | *1999* | 15 | 15-17 | 340 | 0.45 | | 17 | 16-19 | 64 | 0.73 | | 17 | 16-19 | 17 | 0.26 |
|  |  | *2003* | 16 | 15-17 | 354 |  |  | 17 | 15-19 | 81 |  |  | 19 | 17-20 | 47 |  |
|  |  |  |  |  |  |  |  |  |  |  |  |  |  |  |  |  |
| **Urban** | **Males** | *1999* | 17 | 15-19 | 66 | 0.33 | | 17 | 15-20 | 134 | 0.38 | | 18 | 16-21 | 279 | **0.06** |
|  |  | *2003* | 18 | 15-20 | 73 |  |  | 17 | 16-22 | 143 |  |  | 18 | 16-22 | 473 |  |
|  |  |  |  |  |  |  |  |  |  |  |  |  |  |  |  |  |
|  | **Females** | *1999* | 17 | 16-20 | 184 | 0.79 | | 18 | 16-20 | 200 | 0.62 | | 19 | 17-21 | 306 | **0.005** |
|  |  | *2003* | 17 | 16-19 | 192 |  |  | 18 | 17-20 | 198 |  |  | 20 | 18-22 | 554 |  |
|  |  |  |  |  |  |  |  |  |  |  |  |  |  |  |  |  |
