# Supplementary material for: Associations between sexual behaviour change in young people and decline in HIV prevalence in Zambia
Source: BMC Public Health. 2007 Apr 23;7:60. doi: 10.1186/1471-2458-7-60 (PMC1868719; doi:10.1186/1471-2458-7-60)
Supplement: Additional file 14 — Additional table 13. Percentages reporting having had sex by the age of 15 in 1999 and in 2003 based on the age groups of the respondents in 1999. [file 1471-2458-7-60-S14.doc]

**Percentages reporting having had sex by the age of 15 in 1999 and in 2003 based on the age groups of the respondents in 1999.**

|  |  | **Men** | | | | | | **Women** | | | | | |
| --- | --- | --- | --- | --- | --- | --- | --- | --- | --- | --- | --- | --- | --- |
|  |  | **1999** | | **2003** | | | | **1999** | | **2003** | | | |
| **Rural** | **Age in 1999** | **%** | **N** | **%** | **N** | **OR*** | **95% CI** | **%** | **N** | **%** | **N** | **OR*** | **95% CI** |
|  | **15-19** | 42 | 113 | 24 | 156 | **0.45** | **0.25-0.83** | 28 | 176 | 19 | 233 | 0.62 | 0.32-1.19 |
|  | **20-24** | 26 | 141 | 17 | 144 | 0.58 | 0.34-1.02 | 18 | 185 | 21 | 204 | 1.19 | 0.74-1.90 |
|  | **25-29** | 22 | 120 | 13 | 151 | **0.50** | **0.29-0.86** | 19 | 174 | 17 | 147 | 0.88 | 0.53-1.45 |
|  | **30-34** | 23 | 107 | 15 | 98 | 0.59 | 0.22-1.59 | 15 | 144 | 17 | 125 | 1.18 | 0.64-2.19 |
|  | **35-39** | 21 | 87 | 14 | 88 | **0.61** | **0.37-0.98** | 21 | 112 | 22 | 96 | 1.08 | 0.48-2.46 |
|  | **40-44** | 13 | 54 | 13 | 68 | 1.02 | 0.35-2.96 | 17 | 89 | 16 | 90 | 0.91 | 0.49-1.69 |
|  | **45-49** | 10 | 60 | 11 | 45 | 1.12 | 0.30-4.22 | 20 | 54 | 26 | 58 | 1.36 | 0.40-4.61 |
| **Urban** | **15-19** | 26 | 111 | 15 | 284 | 0.52 | 0.27-1.00 | 15 | 157 | 5 | 332 | **0.28** | **0.12-0.65** |
|  | **20-24** | 16 | 195 | 12 | 190 | 0.69 | 0.39-1.20 | 5 | 283 | 4 | 356 | 0.79 | 0.38-1.63 |
|  | **25-29** | 8 | 131 | 6 | 147 | 0.71 | 0.37-1.37 | 6 | 265 | 4 | 245 | 0.78 | 0.37-1.65 |
|  | **30-34** | 10 | 91 | 9 | 91 | 0.88 | 0.45-1.73 | 4 | 163 | 3 | 124 | 0.87 | 0.21-3.69 |
|  | **35-39** | 11 | 56 | 8 | 71 | 0.77 | 0.15-3.83 | 4 | 140 | 1 | 115 | 0.20 | 0.01-3.14 |
|  | **40-44** | 12 | 66 | 6 | 54 | 0.43 | 0.18-1.04 | 8 | 105 | 5 | 86 | 0.59 | 0.20-1.74 |
|  | **45-49** | 5 | 60 | 11 | 46 | 2.32 | 0.80-6.71 | 8 | 51 | 2 | 45 | 0.27 | 0.02-3.26 |

Notes: *The reference is 1999
