# Supplementary material for: Associations between sexual behaviour change in young people and decline in HIV prevalence in Zambia
Source: BMC Public Health. 2007 Apr 23;7:60. doi: 10.1186/1471-2458-7-60 (PMC1868719; doi:10.1186/1471-2458-7-60)
Supplement: Additional file 15 — Additional table 14. HIV prevalence among young people aged 15–24 reporting 'ever' and 'never' sexual activity [file 1471-2458-7-60-S15.doc]

**HIV prevalence among young people aged 15-24 reporting ‘ever’ and ‘never’ sexual activity**

| **Year** | **Response** | **Urban** | | | | | | | | | | | | **Rural** | | | | | | | | | | | |
| --- | --- | --- | --- | --- | --- | --- | --- | --- | --- | --- | --- | --- | --- | --- | --- | --- | --- | --- | --- | --- | --- | --- | --- | --- | --- |
|  |  | **M** | | | | | | **F** | | | | | | **M** | | | | | | **F** | | | | | |
|  |  | **% HIV** | **N** | **Crude OR** | **95% CI** | **AOR** | **95% CI** | **% HIV** | **N** | **Crude OR** | **95% CI** | **AOR** | **95% CI** | **% HIV** | **N** | **Crude OR** | **95% CI** | **AOR** | **95% CI** | **% HIV** | **N** | **Crude OR** | **95% CI** | **AOR** | **95% CI** |
| *1999* | ***Never*** | 6.4 | 155 | Ref. |  | Ref. |  | 7.7 | 220 | Ref. |  | Ref. |  | 4.0 | 25 | Ref. |  | Ref. |  | 5.7 | 53 | Ref. |  | Ref. |  |
|  | ***Ever*** | 7.9 | 277 | 1.25 | 0.55-2.84 | 1.12 | 0.59-2.12 | 23.8 | 421 | **3.72** | **1.83-7.56** | **2.54** | **1.38-4.67** | 8.3 | 241 | 2.17 | 0.28-16.6 | 1.53 | 0.13-18.3 | 10.9 | 329 | 2.05 | 0.89-4.71 | 1.02 | 0.37-2.79 |
| *2003* | ***Never*** | 2.0 | 244 | Ref. |  | Ref. |  | 4.0 | 370 | Ref. |  | Ref. |  | 0 | 68 | Ref. |  | Ref. |  | 0 | 89 | Ref. |  | Ref. |  |
|  | ***Ever*** | 4.0 | 377 | 1.98 | 0.60-6.49 | 1.47 | 0.57-3.76 | 19.2 | 468 | **5.63** | **3.99-7.96** | **4.55** | **2.90-7.14** | 4.2 | 240 | - | - | - | - | 8.4 | 367 | - | - | - |  |
