# Supplementary material for: Associations between sexual behaviour change in young people and decline in HIV prevalence in Zambia
Source: BMC Public Health. 2007 Apr 23;7:60. doi: 10.1186/1471-2458-7-60 (PMC1868719; doi:10.1186/1471-2458-7-60)
Supplement: Additional file 16 — Additional table 15. Age-adjusted odds ratio (AOR) of risk of HIV infection by survey time comparing 2003 and 1995, adjusting for sexual behaviour variables, for urban and rural men and women aged 15–24 with higher education (≥10 years of schooling) [file 1471-2458-7-60-S16.doc]

**Age-adjusted odds ratio (AOR) of risk of HIV infection by survey time comparing 2003 and 1995, adjusting for sexual behaviour variables, for urban and rural men and women aged 15-24 with higher education (≥10 years of schooling)**

**a)**

| **Urban men aged 15-24 with 10+ years of school** | | | | |
| --- | --- | --- | --- | --- |
| One by one behaviour indicator included | | | | |
| **Behaviour indicator** | **Chi square** | **Chi-square change** | **AOR** | **95% CI** |
| Number of sex partners past 12 months | **8.94** | **-17.06** | **0.52** | **0.29-0.94** |
| Casual partner past year | **7.49** | **-18.51** | **0.53** | **0.26-1.08** |
| Condom use at last sex | **3.48** | **-22.52** | **0.45** | **0.23-0.88** |
| Ever used condom | **14.69** | **-11.31** | **0.49** | **0.28-0.85** |
| Condom use with last casual partner** | **3.48** | **-22.52** | **0.63** | **0.23-1.72** |
| STI last year | 28.01 | 2.01 | 0.42 | 0.29-0.59 |

Notes: **Before adding the behaviour variables the Chi square was 26.00 and AOR 0.41 (0.28-0.59) in 2003 compared to 1995.** If the chi square changed > |3.84|, the added variable was a confounding variable. The denominator was the number of sexually active respondents last year. . **The chief confounder in this step.

b)

| **Urban women aged 15-24 with 10+ years of school** | | | | | | | | | | | | | | |
| --- | --- | --- | --- | --- | --- | --- | --- | --- | --- | --- | --- | --- | --- | --- |
| One by one behaviour indicator included | | | | | Two behaviour variables included | | | | | Three behaviour variables included | | | | |
| **Beh. indicator** | **Chi-square** | **Chi-square change** | **AOR** | **95% CI** | **Beh. indicator** | **Chi-square** | **Chi-square change** | **AOR** | **95% CI** | **Beh. indicator** | **Chi-square** | **Chi-square change** | **AOR** | **95% CI** |
| Ever given birth | **22.35** | **-18.82** | **0.37** | **0.23-0.61** | Frequent dry sex and ever given birth | 13.64 | -1.78 | 0.51 | 0.31-0.84 | Frequent dry sex and number of partners and ever given birth | 4.95 | -1.78 | 0.54 | 0.31-0.93 |
| Number of sex partners past 12 months | **13.33** | **-27.84** | **0.42** | **0.24-0.73** | Frequent dry sex and number of partners** | **6.73** | **-8.69** | **0.50** | **0.29-0.86** | - |  |  |  |  |
| Casual partner past year | **15.10** | **-26.07** | **0.40** | **0.22-0.70** | Frequent dry sex and casual partner last year | 13.44 | -1.98 | 0.48 | 0.27-0.83 | Frequent dry sex and number of partners and casual partner | 10.56 | 3.83 | 0.49 | 0.28-0.85 |
| Condom use at last sex | **18.23** | **-22.94** | **0.39** | **0.25-0.61** | Frequent dry sex and condom use at last sex | **9.31** | **-6.11** | **0.46** | **0.31-0.70** | Frequent dry sex and number of partners and condom use at last sex | 5.06 | -1.67 | 0.49 | 0.33-0.73 |
| Ever used condom | **14.49** | **-26.68** | **0.39** | **0.22-0.70** | Frequent dry sex and ever condom use | **6.82** | **-8.6** | **0.48** | **0.26-0.89** | Frequent dry sex and number of partners and ever condom use | 5.58 | -1.15 | 0.49 | 0.27-0.92 |
| Frequent dry sex** | **15.42** | **-25.75** | **0.45** | **0.28-0.72** | - |  |  |  |  |  |  |  |  |  |

Notes: Before adding the behaviour variables the Chi square was 41.17 and AOR 0.27 (0.17-0.43) in 2003 compared to 1995**.** If the chi square changed > |3.84|, the added variable was a confounding variable. The denominator was the number of sexually active respondents last year.

c)

| **Rural men aged 15-24 with 10+ years of school** | | | | | | | | | |
| --- | --- | --- | --- | --- | --- | --- | --- | --- | --- |
| One by one behaviour indicator included | | | | | Two behaviour variables included | | | | |
| **Beh. indicator** | **Chi-square** | **Chi-square change** | **AOR** | **95% CI** | **Beh. indicator** | **Chi-square** | **Chi-square change** | **AOR** | **95% CI** |
| Number of sex partners past 12 months | **20.74** | **-172.99** | **0.35** | **0.13-0.90** | Casual partner last year and number of sexual partners* | 29854.28 | -39370 | 0.29 | 0.20-0.42 |
| Casual partner past year** | **69224.28** | **69030.55** | **0.60** | **0.49-0.75** | - |  |  |  |  |
| Condom use at last sex | **8022.54** | **7828.81** | **0.39** | **0.23-0.65** | Casual partner past year and condom use at last sex** | **-** | **-** | **0.70** | **0.49-0.99** |
| Ever used condom | **1704.62** | **1510.89** | **0.25** | **0.16-0.38** | Casual partner past year and ever condom use* | 165481.25 | 96256.97 | 0.53 | 0.35-0.81 |
| Condom use with last casual partner | **-** | **-** | **-** | **-** | Casual partner past year and condom use with last casual partner | - | - | - | - |
| STI past year | 193.73 | 0 | 0.24 | 0.13-0.42 | Casual partner past year and STI past year | 69224.28 | 0 | 0.60 | 0.49-0.75 |

Notes: Before adding the behaviour variables the Chi square was 193.73 and AOR 0.24 (0.13-0.42) in 2003 compared to 1995. If the chi square changed > |3.84|, the added variable was a confounding variable. The denominator was the number of sexually active respondents last year. *The Chi square change was significant, but the AOR was further from 1, which meant that adjusting for ‘condom use at last sexual intercourse’ or ‘number of sexual partners past year’ in addition to ‘any casual partners past year’ increased the strength of the association between HIV and survey time, rather than reducing it.

d)

| **Rural women aged 15-24 with 10+ years of school** | | | | |
| --- | --- | --- | --- | --- |
| One by one behaviour indicator included | | | | |
| **Beh. indicator** | **Chi-square** | **Chi-square change** | **AOR** | **95% CI** |
| Ever given birth | **55.12** | **-18.87** | **0.20** | **0.02-1.64** |
| Number of sex partners last 12 months | **23.90** | **-50.09** | **0.22** | **0.04-1.16** |
| Casual partner last year | **28.01** | **-45.98** | **0.21** | **0.06-0.78** |
| Condom use at last sex | **-** |  |  |  |
| Ever used condom** | **198.82** | **124.83** | **0.76** | **0.45-1.29** |
| Frequent dry sex | **41.94** | **-32.05** | **0.27** | **0.14-0.52** |

Notes: Before adding the behaviour variables the Chi square was 73.99 and AOR 0.18 (0.04-0.85) in 2003 compared to 1995**.** If the chi square changed > |3.84|, the added variable was a confounding variable. The denominator was the number of sexually active respondents last year.
