# Supplementary material for: Associations between sexual behaviour change in young people and decline in HIV prevalence in Zambia
Source: BMC Public Health. 2007 Apr 23;7:60. doi: 10.1186/1471-2458-7-60 (PMC1868719; doi:10.1186/1471-2458-7-60)
Supplement: Supplementary file 16 [file 1471-2458-7-60-S13.doc]

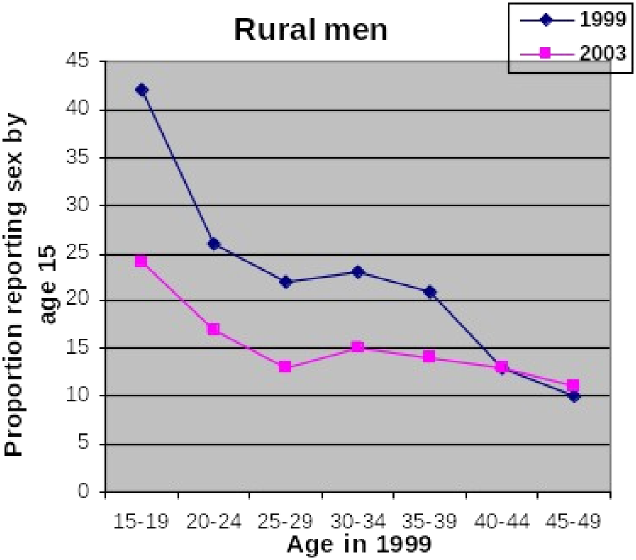
Rural men
1999
2003
Proportion reporting sex by age 15
0
5
10
15
20
25
30
35
40
45
15-19
20-24
25-29
30-34
35-39
40-44
45-49
Age in 1999

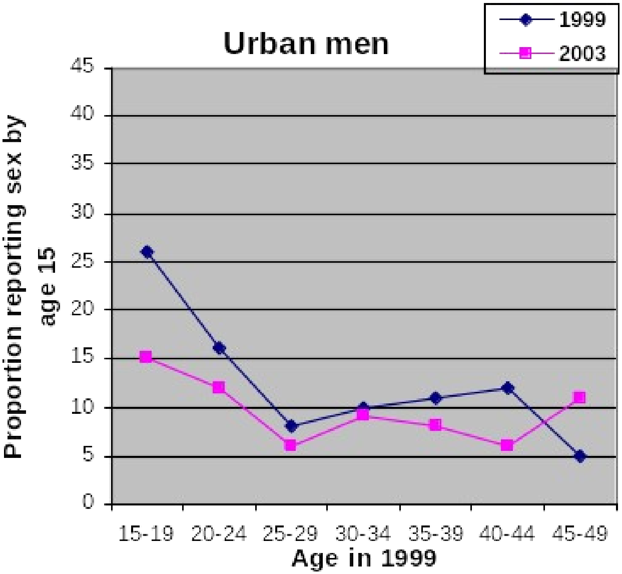
Urban men
1999
2003
Proportion reporting sex by age 15
0
5
10
15
20
25
30
35
40
45
15-19
20-24
25-29
30-34
35-39
40-44
45-49
Age in 1999

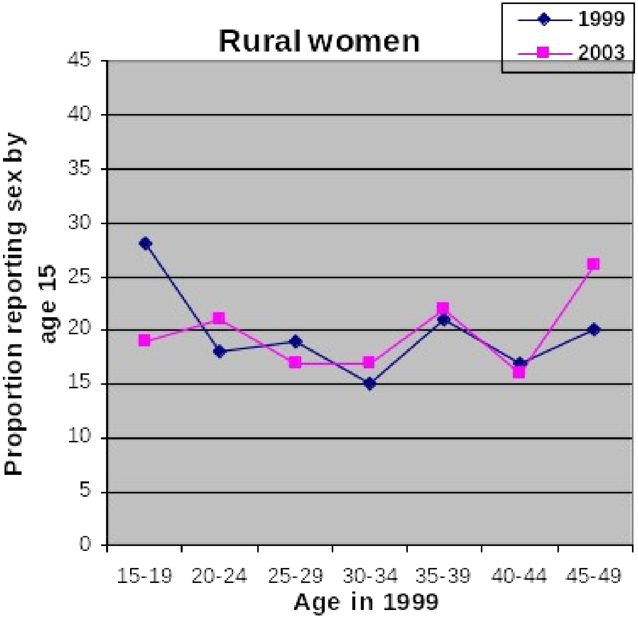
Rural women
1999
2003
Proportion reporting sex by age 15
0
5
10
15
20
25
30
35
40
45
15-19
20-24
25-29
30-34
35-39
40-44
45-49
Age in 1999

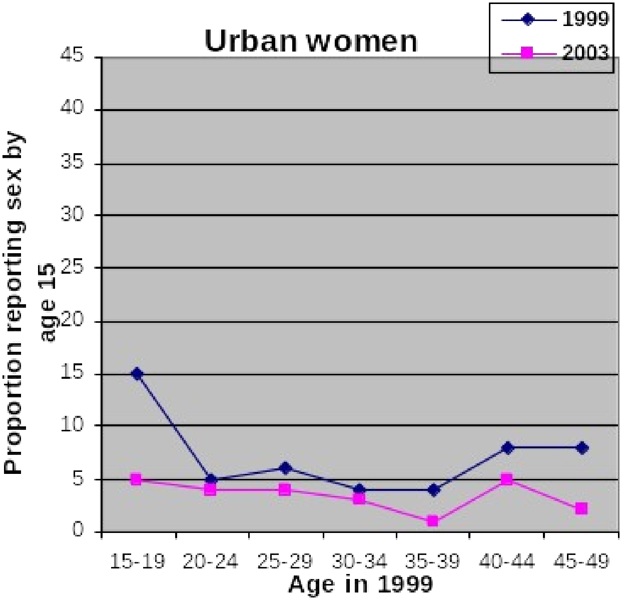
Urban women
1999
2003
Proportion reporting sex by age 15
0
5
10
15
20
25
30
35
40
45
15-19
20-24
25-29
30-34
35-39
40-44
45-49
Age in 1999
