# Supplementary material for: Associations between sexual behaviour change in young people and decline in HIV prevalence in Zambia
Source: BMC Public Health. 2007 Apr 23;7:60. doi: 10.1186/1471-2458-7-60 (PMC1868719; doi:10.1186/1471-2458-7-60)
Supplement: Supplementary file 17 [file 1471-2458-7-60-S17.doc]

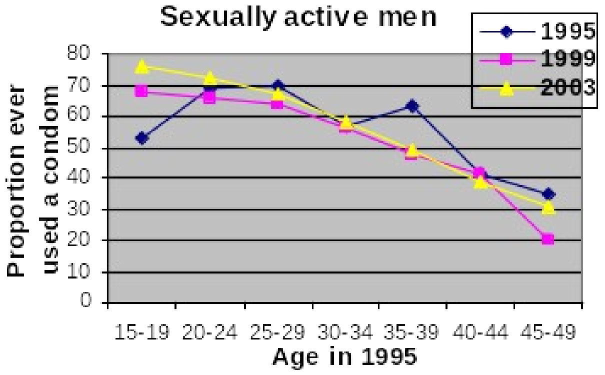
Sexually active men
1995
1999
2003
Proportion ever used a condom
0
10
20
30
40
50
60
70
80
15-19
20-24
25-29
30-34
35-39
40-44
45-49
Age in 1995

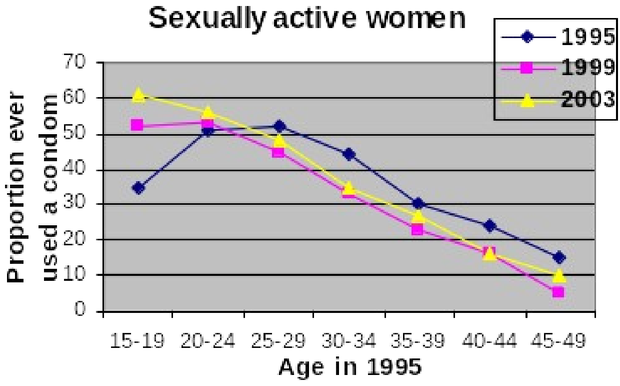
Sexually active women
1995
1999
2003
Proportion ever used a condom
0
10
20
30
40
50
60
70
15-19
20-24
25-29
30-34
35-39
40-44
45-49
Age in 1995
