# Supplementary material for: Associations between sexual behaviour change in young people and decline in HIV prevalence in Zambia
Source: BMC Public Health. 2007 Apr 23;7:60. doi: 10.1186/1471-2458-7-60 (PMC1868719; doi:10.1186/1471-2458-7-60)
Supplement: Supplementary file 18 [file 1471-2458-7-60-S18.doc]

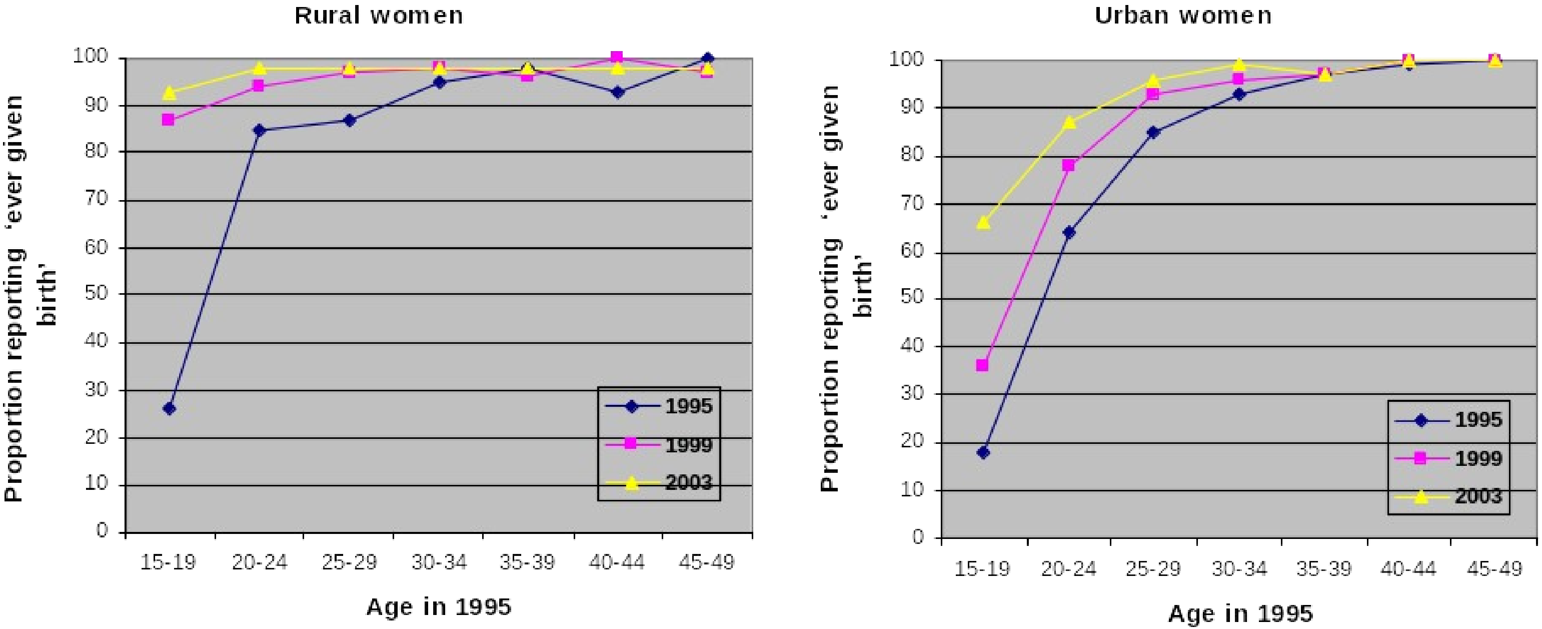

Rural women
Proportion reporting 'ever given birth'
100
90
80
70
60
50
40
30
20
10
0
15-19
20-24
25-29
30-34
35-39
40-44
45-49
Age in 1995
1995
1999
2003
Urban women
Proportion reporting 'ever given birth'
100
90
80
70
60
50
40
30
20
10
0
15-19
20-24
25-29
30-34
35-39
40-44
45-49
Age in 1995
1995
1999
2003
